# Personality growth after relationship losses: Changes of perceived control in the years around separation, divorce, and the death of a partner

**DOI:** 10.1371/journal.pone.0268598

**Published:** 2022-08-03

**Authors:** Eva Asselmann, Jule Specht

**Affiliations:** 1 Faculty of Health, HMU Health and Medical University, Potsdam, Germany; 2 Department of Psychology, Humboldt-Universität zu Berlin, Berlin, Germany; Max Planck Institute for Human Development, GERMANY

## Abstract

**Background:**

Previous research suggests that romantic relationships play a crucial role for perceived control. However, we know surprisingly little about changes in perceived control before and after the end of romantic relationships.

**Methods:**

Based on data from the Socio-Economic Panel Study (SOEP), a nationally representative household panel study from Germany, we examined changes of perceived control in the years around separation from a partner (*N =* 1,235), divorce (*N =* 423), and the death of a partner (*N =* 437).

**Results:**

Multilevel analyses revealed that external control beliefs were higher in but not beyond the first year after separation from a partner. Internal and total control beliefs increased gradually in the years after separation. Moreover, internal control beliefs were higher in and especially beyond the first year after the death of a partner compared to the years before. No evidence was found that perceived control already changed in the years before relationship losses or in the years around a divorce.

**Conclusion:**

Taken together, these findings point toward stress-related growth of perceived control after some relationship losses–especially separation and the death of a partner.

## Introduction

Maintaining control is a basic human need [1, 2]. However, humans differ in their perceived control [3], and this impacts how well they feel [4, 5], how healthy they are [6–8], and how they master the challenges they are faced with [1, 2, 9, 10].

Perceived control not only affects how we deal with our life but is also influenced by our life experiences, including social and romantic relationships [2]. This study examined how perceived control changes in the years around romantic relationship losses, including separation, divorce, and the death of a partner.

### Perceived control

Perceived control can be seen as a personality trait and refers to generalized beliefs about what determines one’s own life [11, 12]. Individuals with high internal control beliefs are convinced to be able to influence their environment and their future by their own behavior. Conversely, people with high external control beliefs are convinced that things that happen to them largely result from external factors such as powerful others, luck, chance, or fate.

Internal and external control beliefs can be conceptualized as one continuum (with low perceived control indicating low internal control beliefs and high external control beliefs, and high perceived control indicating high internal control beliefs and low external control beliefs) or two separate dimensions [12]. Whether internal and external control beliefs lay on opposite pols of one dimension or are two separate dimensions has been discussed controversially [12, 13]. Some studies found a one-factor solution but others a two-factor solution for internal and external control beliefs [4, 14, 15], which suggests that individuals with high internal control beliefs not necessarily have low external control beliefs and vice versa.

According to Motivational Theory of Lifespan Development [1], maintaining a sense of control is a key feature of adaptive capacity, motivation regulation, and successful development across the lifespan. Consistently, higher perceived control has been associated with favorable developmental outcomes [2, 12], including higher subjective well-being [4, 5], better mental and physical health [6–8], as well as lower mortality [16–18].

Perceived control not only relates to developmental outcomes but also develops itself. Developmental research found that perceived control increased in young adulthood, remained relatively stable in middle adulthood, and decreased in old age [3, 5, 19–21].

In line with Motivational Theory of Lifespan Development [1], such age-graded changes in perceived control might relate to age-graded opportunities and constraints to shape one’s life. Younger adults typically experience many gain-based events (e.g., the start of a romantic relationship or working life) that can be influenced by their own behavior. In contrast, older adults tend to experience a higher proportion of losses (e.g., health impairments or bereavement) that cannot be avoided [22, 23]. These aged-graded changes in gains and losses might explain the perceived control trajectory across the lifespan.

### Romantic relationships and perceived control

Perceived control relates to social experiences [24, 25]. Social relationships signal stability, security, and predictability and provide humans with instrumental and emotional resources of social support. Self-Efficacy Theory posits that positive social interactions and encouragement from others (e.g., social modeling and verbal persuasion) are a major source of perceived control [25]. Consistently, social participation, social activities, and subjective social support have been associated with higher perceived control [7, 16, 26, 27], whereas loneliness has been associated with lower perceived control [27, 28].

In adolescence and adulthood, romantic relationships increase in importance. In the domain of love, not only gain-based events (e.g., starting a relationship and getting married) but also loss experiences (e.g., separation, divorce, and the death of a partner) have been associated with changes in core personality traits, including the Big Five [29–31], subjective well-being [31–37], and self-esteem [38, 39].

Previous research has shown that perceived control was associated with higher relationship satisfaction [26] and that perceived control increased during the first years of marriage [40]. However, the role of relationship losses for differences and changes in perceived control remains largely unresolved.

#### Theoretical perspectives

Motivational Theory of Lifespan Development [1] assumes that humans seek to actively shape their life and have remarkable capacities to maintain high levels of perceived control, even when it comes to losses. The theory distinguishes between primary and secondary control strategies. Primary control strategies aim at changing the environment in line with personal goals, whereas secondary control strategies aim at changing the self in line with the environment. After a breakup, people might, for example, try to win back their ex-partner (primary control strategy) or to disengage from their ex-partner (secondary control strategy) to regain control.

According to Motivational Theory of Lifespan Development, especially secondary control strategies are important after losses [1]. Losses mean that certain primary control goals (e.g., spending the future with a partner) become unattainable. Secondary control strategies serve to disengage from these goals, to protect one’s motivational resources, and to set new attainable goals, supporting primary control in the long run. After a breakup, people might, for example, blame their ex-partner or attribute their loss to another external source (e.g., a love affair). Using secondary control strategies might help them to disengage from their ex-partner, uphold their self-esteem, as well as set and attain new primary control goals (e.g., finding a new partner). Taken together, this suggests that individuals might switch to secondary control strategies and have lower perceived control (i.e., lower internal control beliefs and higher external control beliefs) in the first months after relationship losses. In the long run, however, they might switch back to primarily control strategies, leading to a regain of perceived control.

Set-Point Theory [33] posits that humans have a set-point on specific personality traits and that their trait levels fluctuate around this point over time. For example, subjective well-being might drop shortly after relationship losses but bounce back to its set-point in the long run. Consistently, relationship losses (e.g., separation, divorce, and the death of a partner) have been associated with a decrease of subjective well-being [31–37] and self-esteem [38, 39] in the surrounding years. However, in most cases, this decline was transient and attenuated in the long run. Taken together, perceived control might be lower in the short run but not in the long run after relationship losses.

Research on stress-related growth suggests that humans might grow from adverse experiences and gain inner strength and maturity after relationship losses [41–43]. According to this idea, perceived control might not merely bounce back but even grow beyond its pre-loss levels in the long run.

In line with Theory of Learned Helplessness [44], however, it is more plausible that perceived control decreases permanently after relationship losses. Loss experiences (e.g., the death of a partner) often create a situation that cannot or can only partly be influenced by one’s own behavior. This might trigger strong feelings of uncontrollability and helplessness and long-term impairments of perceived control. From this point of view, perceived control might be lower not only in but also beyond the first year after relationship losses than before.

Taken together, perceived control might be lower in the first year after relationship losses than before. However, different theories lead to different predictions with respect to long-term changes after such experiences. In line with Theory of Learned Helplessness, perceived control might continue to be lower more than one year after relationship losses. In line with Set-Point Theory, perceived control might bounce back to its pre-loss levels. As suggested by research on stress-related growth, perceived control might even grow beyond its pre-loss levels in the following years.

#### Cross-sectional findings

A series of cross-sectional studies examined associations between relationship status and perceived control. They found that perceived control was higher in individuals who had versus had not separated from a partner in the past [45, 46]. Specifically, Doherty [45] demonstrated that perceived control (conceptualized as one continuum from external to internal) was higher in divorced versus never married, married, or widowed individuals (who did not differ from each other in their perceived control). Ross [46] found that perceived control was higher in single and divorced versus married women and in divorced versus married men.

Taken together, these findings suggest that perceived control might grow after relationship losses. However, based on these cross-sectional findings, one could also speculate whether individuals with higher perceived control are simply more likely to break up (selection effects).

#### Longitudinal findings

Few longitudinal studies investigated the role of relationship losses for changes in perceived control. In contrast to cross-sectional findings, they suggest that perceived control might be lower after relationship losses than before [47, 48]. However, longitudinal findings on changes in perceived control around the death of a partner are rare and inconclusive—possibly because large samples are required to study nuanced changes in perceived control around this rare experience.

Nowicki and colleagues [47] prospectively followed up expectant parents from the ALSPAC study over six years and investigated the role of relationship losses during this time for changes in perceived control (conceptualized as one continuum from external to internal). During pregnancy and six years later, they distinguished between women and men with low versus high perceived control, respectively. They found that women who separated from a partner were less likely to change from low to high perceived control and more likely to change from high to low perceived control over time. Similarly, women who got divorced were more likely to change from high to low perceived control. In men, separation and divorce were unrelated to changes in perceived control. Neither in women nor in men, the death of a friend or relative was associated with changes in perceived control.

Using data from a nationally representative household panel study from Australia (HILDA study), Cobb-Clark and Schurer [21] investigated whether changes in perceived control (conceptualized as one continuum from external to internal) over up to four years differed between individuals who did versus did not separate from a partner or experience the death of a partner/child during this time frame. In this study, relationship losses were unrelated to changes in perceived control.

Finally, Doherty [48] investigated changes in perceived control (conceptualized as one continuum from external to internal) over three waves (eight years) in initially married women who were married throughout the study or got divorced until first follow-up. Compared to women who stayed married, women who got divorced until first follow-up (three years after baseline) experienced a stronger decrease of perceived control until this time point. However, perceived control was comparable between both groups at baseline and last follow-up (eight years after baseline). In line with Motivational Theory of Lifespan Development, Set-Point Theory, and Theory of Learned Helplessness, these findings suggest that perceived control might be lower shortly after relationship losses. Consistent with Set-Point Theory, they further support the idea that perceived control might bounce back to its pre-loss levels over longer periods of time.

### Methodological challenges

Studying the role of relationship losses for perceived control requires considering differences between individuals who will and will not experience these losses in the following years (selection effects). On the one hand, individuals with higher perceived control might have more stable romantic relationships, be more satisfied with their relationship, and feel more capable to actively shape and improve their relationship [26]. Thus, they might be less likely to break up. On the other hand, individuals with higher perceived control might feel more capable to manage their life without a partner. Thus, they might be more likely to end their relationship in times of trouble [45, 46].

Over and above selection effects, perceived control might differ between individuals who have or have not experienced relationship losses in the previous years (post-loss differences). In line with different theoretical assumptions on changes in perceived control around relationship losses, perceived control might be lower (Theory of Learned Helplessness), comparable (Set-Point Theory), or higher in individuals with versus without loss experiences in the past.

Furthermore, perceived control might vary at different junctions around relationship losses. In line with different theoretical perspectives (see above), the impact of relationship losses might unfold gradually over time. That is, the data of the actual loss often marks the peak of a change trajectory that sets in much earlier and continues beyond this date. Before a breakup, people often experience increased relationship distress and potentially try hard to fix or ignore their problems [30, 39]. Before a partner dies, she/he is often ill, requires intense care, or is predicted to die in the near future [29, 49]. After their loss, many people not only feel depressed or lonely but also have to deal with organizational tasks and, for example, split their household, resolve financial and legal issues, or adjust their family and working life to the new situation. In other words, losing a partner might relate to different psychological challenges in the years around this experience. Thus, gradual changes of perceived control in the years before (anticipation effects) and after (socialization effects) relationship losses as well as short- and long-term effects in and beyond the first year after these experiences need to be considered.

### Gender and age differences

In terms of gender, women might experience a higher short-term decrease but also higher long-term increase of perceived control after relationship losses than men [41, 42]. Especially in women, relationship losses have been associated with differences and changes in perceived control [46–48]. Although traditional gender roles have become less important, women might tend to focus more on their partner and family than men. Thus, they might experience a stronger decrease of perceived control when their relationships end [46]. At the same time, women might tend to more actively cope with relationship dissolution and grief than men and, for example, more strongly engage in (other) social relationships thereafter [50].

In terms of age, it is plausible to assume that relationship losses, especially the death of a partner, might have more detrimental effects on younger versus older individuals. Compared to older individuals, younger individuals might be more flexible, energetic, and socially active and thus be able to adjust to a new situation without a partner more easily [36, 51]. On the other hand, older (and more experienced) individuals might have already developed a wider range of (emotion-focused) coping strategies [52, 53]. Such strategies might be particularly useful to cope with the death of a partner, an extremely stressful event that predominantly occurs in old age.

### Aims

This study aimed to examine associations between major relationship losses (separation, divorce, and the death of a partner) and perceived control and to test whether these associations vary by gender and age. We used data from the SOEP (*N* = 14,772), a nationally representative household panel study from Germany with ongoing yearly assessments since 1984. In the SOEP, participants were yearly asked whether they had separated from a partner, gotten divorced, or experienced the death of a partner in the current or previous year. perceived control was measured repeatedly with the same questionnaire in 1994, 1995, and 1996.

### Hypotheses

Based on different theoretical perspectives and empirical evidence reviewed above, we had the following–partly contradictory–hypotheses:

*Selection effects*

H1. Perceived control **is lower** in individuals who will (versus will not) experience the respective loss in the following years [26].
H2. Perceived control **is higher** in individuals who will (versus will not) experience the respective loss in the following years [45, 46].

*Post-loss differences*

H3. Perceived control **is lower** in individuals who have (versus have not) experienced the loss in the previous years (Theory of Learned Helplessness) [44].
H4. Perceived control **does not differ** between individuals who have (versus have not) experienced the respective loss in the previous years (Set-Point Theory) [33].
H5. Perceived control **is higher** in individuals who have (versus have not) experienced the respective loss in the previous years (stress-related growth) [41–43].

*Short-term effects*

H6. Perceived control **is lower** in the first year after the respective loss versus all other years (Motivational Theory of Lifespan Development; [1] Set-Point Theory; [33]; and Theory of Learned Helplessness; [44]).

*Socialization effects*

H7. Perceived control **increases gradually** in the years after the respective loss (Motivational Theory of Lifespan Development; [1] stress-related growth; [41–43]).

*Long-term effects*

H8. Perceived control **is lower** more than one year after the respective loss versus before (Theory of Learned Helplessness) [44].
H9. Perceived control **does not differ** more than one year after the respective loss versus before (Set-Point Theory) [33].
H10. Perceived control **is higher** more than one year after the respective loss versus before (stress-related growth) [41–43].

*Gender differences*

H11. The hypothesized short-term effects on perceived control are **stronger in women** versus men [41, 42].

*Age differences*

H12. The hypothesized short-term effects on perceived control are **stronger in younger** versus older individuals [52, 53].

Moreover, we explored the possibility of other changes in perceived control around the respective loss (e.g., anticipation effects). To account for a one- and two-factor solution of perceived control, we conducted the analyses for internal, external, and total control beliefs (with higher scores indicating higher internal control beliefs and lower external control beliefs, respectively).

## Materials and methods

### Study sample

We used data from the Socio-Economic Panel Study (SOEP), a nationally representative household panel study from Germany with multistage probability sampling. Data are collected yearly since 1984 (ongoing) and mostly stem from face-to-face interviews with all adult members of the participating households.

The initial sample from 1984 was regularly replenished with refreshment cohorts. This was done to counteract attrition, to increase the overall sample size, and to allow for detailed analyses of specific sub-samples. Because refreshment cohorts entered the panel in different years, not all participants provided data on relationship losses and perceived control in each year.

Further information on the SOEP, including the sample structure, subsamples, and panel attrition, has been previously presented [54, 55] and can be found at https://paneldata.org/soep-core. All procedures, measures, variables, and their coding can be found at https://paneldata.org/soep-core. A collection of previous SOEP publications can be found at https://www.diw.de/sixcms/detail.php?id=diw_02.c.298578.en. The SOEP data are available from the DIW Berlin after signing a data distribution contract (https://www.diw.de/en/diw_02.c.222829.en/access.html). Because the SOEP data are personal data, which are subject to special protections in Europe, a data distribution contract is mandatory to access the data. After signing the contract, the data can be accessed by others in the same manner by which the authors obtained them. The analytic code is attached as supplemental material (S1 File). The analyses were not pre-registered.

The authors assert that all procedures contributing to this work comply with the ethical standards of the relevant national and institutional committees on human experimentation and with the Helsinki Declaration of 1975, as revised in 2013. Because the study only involved secondary analyses of anonymized SOEP data from the DIW Berlin, obtaining ethical approval from an IRB or ethics committee was not required.

### Assessment of relationship losses

Participants were yearly asked whether and when (year and month) they had separated from a partner, gotten divorced, or experienced the death of a partner in the current or previous year.

### Assessment of perceived control

Perceived control was assessed in 1994, 1995, and 1996 with eight items based on the scale by Rotter [11]. Internal control beliefs were captured with three and external control beliefs with five items, labeled from 1 = ‘applies completely’ to 4 = ‘does not apply’. We reversed inverted items, so that higher scores reflected higher internal control beliefs or higher external control beliefs, respectively.

For this questionnaire, a clear two-factor solution has been found for internal and external control beliefs [4]. Thus, we treated internal and external control beliefs as two separate dimensions. However, because perceived control has often been conceptualized as a single bipolar dimension [21, 45–48, 56, 57], we additionally considered total control beliefs. We revised the items for external control beliefs for the total score, so that lower total control beliefs scores indicate lower internal control beliefs/higher external control beliefs and higher total control beliefs scores indicate higher internal control beliefs/lower external control beliefs. Across all three waves, internal consistencies in our sample (*N* = 14,772) were *α* = .56 for internal control beliefs, *α* = .77 for external control beliefs, and *α* = .72 for total control beliefs. The correlation between internal and external control beliefs was *r* = -.21.

### Statistical analysis

Stata 15 [58] was used for the analyses. The analytic codes are attached as supplemental material (S1 File).

Participants who provided any perceived control data in 1994, 1995, or 1996 (*N* = 14,772) were considered. We distinguished between individuals who did versus did not separate from a partner, get divorced, or experience the death of a partner between 1991 (three years prior to the first perceived control assessment in 1994) and 1999 (three years after the last perceived control assessment in 1996), respectively. In participants who experienced the respective loss between 1991 and 1999, we coded the date of this experience relative to the date of the respective perceived control assessment in 1994, 1995, and 1996 (in years and months). In our multilevel approach, we combined within- and between-person information. Because participants experienced the respective loss at different time points, this approach allowed us to obtain fine-grained data on perceived control from five years before (in participants who provided perceived control data in 1994 and experienced the respective loss in 1999) to five years after the respective loss (in participants who experienced the respective loss in 1991 and provided perceived control data in 1996).

In participants who repeatedly experienced the same loss between 1991 and 1999, we considered their earliest loss during this time frame. We also considered whether individuals had or had not experienced the respective loss prior to our study period (past-loss effects, see below).

Similar to Denissen and colleagues [31] as well as Asselmann and Specht [29, 30, 59], we used multilevel analyses with measurement occasions (Level 1) nested within persons (Level 2) nested within households (Level 3), built separate models per loss and outcome, and modeled the effects as fixed effects.

First, we considered all individuals who provided perceived control data in 1994, 1995, or 1996 (N = 14,772) to examine differences in perceived control between individuals who did and did not experience the respective loss between 1991 and 1999 (selection and post-loss difference effects). Specifically, we simultaneously regressed the standardized score for internal, external, and total control beliefs, respectively, on gender, linear, quadratic, and cubic age, a testing variable (to account for effects due to repeated perceived control assessments), a past-loss variable (to consider effects due to past experiences of the respective loss prior to 1991), and a selection/post-loss difference variable. This categorical selection/post-loss difference variable was coded with 0 in participants who did not experience the respective loss between 1991 and 1999. In participants who experienced the respective loss between 1991 and 1999, it was coded with 1 for perceived control assessments before this experience and with 2 for perceived control assessments after this experience. We compared category 1 versus 0 to examine selection effects and category 2 versus 0 to examine post-loss differences. Table 1 summarizes how each variable was defined and coded. Examples for the coding are given in S1 Table.

**Table 1:** Description and coding of the included predictors.

Control variables in all analyses (based on the total sample and subsamples of individuals who experienced the respective loss between 1991 and 1999)		
Gender (Level 2 ^a^)	• Gender effects	• Coded with 0 for females • Coded with 1 for males • Grand-mean centered (in the total sample)
Linear age (Level 1)	• Linear age effects	• Age (in years) at the respective assessment of perceived control • Grand-mean centered (in the total sample) • The linear age variable was divided by 10 to be able to display even small effects rounded at two decimals
Quadratic age (Level 1)	• Quadratic age effects	• Linear age variable ^2^
Cubic age (Level 1)	• Cubic age effects	• Linear age variable ^3^
Testing (Level 1)	• Effects due to repeated assessments of perceived control	• Coded with 0 for the first assessment of perceived control • Coded with 1 for the second assessment of perceived control • Coded with 2 for the third assessment of perceived control • Grand-mean centered (in the total sample)
Past-loss (Level 2 ^a^)	• Effects due to experiences of the respective loss prior to our study period (before 1991)	• Coded with 1 in individuals who experienced the respective loss before 1991 • Coded with 0 in individuals who did not experience the respective loss before 1991 • Grand-mean centered (in the total sample)
Analyses in the total sample (to examine differences in perceived control between individuals with and without respective loss)		
Selection and post-loss difference (Level 1)	• Differences in perceived control between individuals who did not experience the respective loss between 1991 and 1990 and individuals who experienced the respective loss *after* the respective assessment of perceived control (selection effects, category 1 versus 0) • Differences in perceived control between individuals who did not experience the respective loss between 1991 and 1990 and individuals who experienced the respective loss *before* the respective assessment of perceived control (post-loss difference effects, category 2 versus 0)	• Coded with 0 in individuals who did not experience the respective loss between 1991 and 1999 • Coded with 1 for assessments of perceived control *before* the respective loss in individuals who experienced this loss until 1999 • Coded with 2 for assessments of perceived control *after* the respective loss in individuals who experienced this loss in or after 1991
Analyses in individuals who experienced the respective loss between 1991 and 1999 (to indicate changes in perceived control before and after the respective loss)		
Anticipation (Level 1)	• Linear changes of perceived control in the years before the respective loss	• Coded with the time span (in years and months) between the respective assessment of perceived control and the respective loss for all assessments of perceived control *before* this experience • Coded with 0 for assessments of perceived control *after* the respective loss
Socialization (Level 1)	• Linear changes of perceived control in the years after the respective loss	• Coded with the time span (in years and months) between the respective assessment of perceived control and the respective loss for all assessments of perceived control *before* this experience • Coded with 0 for assessments of perceived control *before* the respective loss
Short-term (Level 1)	• Short-term changes of perceived control in the first year after the respective loss (compared to all other years)	• Coded with 1 for assessments of perceived control in the first year after the respective loss • Coded with 0 for all other assessments of perceived control
Long-term (Level 1)	• Long-term changes of perceived control more than one year after the respective loss (compared to all previous years)	• Coded with 1 for assessments of perceived control more than one year after the respective loss • Coded with 0 for all other assessments of perceived control

*Note*.

^a^ Level 2 variables had the same values for each observation (i.e., in 1994, 1995, and 1996) of the same person at Level 1.

Second, we only considered individuals who separated from a partner (*N* = 1,235), got divorced (*N* = 423), or experienced the death of a partner (*N* = 437) between 1991 and 1999 to examine loss-related changes in perceived control. Specifically, we simultaneously regressed the standardized score for internal, external, and total control beliefs, respectively, on gender, linear, quadratic, and cubic age, the testing variable, the past-loss variable, and four time-dependent variables (anticipation, socialization, short-term, and long-term). These time-dependent variables coded how the date of the respective loss was temporarily related to the date of the respective perceived control assessment and were used to model anticipation, socialization, short-term, and long-term effects (see Table 1 and S1 Table for more details).

To test for interactions with gender and age, we repeated our main analyses and added interaction terms between the respective predictor (i.e., the selection/post-loss difference, anticipation, socialization, short-term, and long-term variable) and gender or age, respectively. Each interaction was tested separately to avoid multicollinearity. Significant interactive effects were decomposed to assess their direction. That is, we built the respective model separately in women and men or in younger and older individuals (grand-mean split).

The alpha level was set at .05. The main analyses on changes in perceived control around relationship losses refer to three losses (separation, divorce, and the death of a partner) * three outcomes (internal, external, and total control beliefs) * four time-dependent effects (anticipation, socialization, short-term, and long-term). We did not adjust for multiple testing because each effect relates to another research question/hypothesis. However, researchers who believe that adjustment for multiple testing is necessary may refer to this total number of main effects.

## Results

### Sample characteristics

Frequencies and percentages of participants who did and did not experience the respective loss between 1991 and 1999 and provided perceived control data in 1994, 1995, and/or 1996 are shown in Table 2, which also includes information on the overall number of assessments. Sample characteristics for these groups are presented in Table 3.

**Table 2:** Frequencies and percentages of individuals who did and did not experience the respective loss between 1991 and 1999 and provided information on perceived control in 1994, 1995, and/or 1996 (including information on the overall number of assessments; *N* = 14,772).

	Assessment of perceived control			
	1994	1995	1996	Number of assessments
Sample per event	*N (%)*	*N (%)*	*N (%)*	*M (SD)*
Total sample (*N* = 14,772)	11,167	11,676	13,472	2.46
	(75.59)	(74.04)	(91.19)	*(*0.82)
Separation				
No separation (*N* = 13,537)	10,145	10,599	12,317	2.44
	(74.94)	(78.29)	(90.98)	(0.82)
Separation (*N* = 1,235)	1,022	1,077	1,155	2.63
	(82.75)	(87.21)	(93.52)	(0.68)
in 1991 (*N* = 102)	93	92	91	2.71
	(91.18)	(90.20)	(89.22)	(0.65)
in I992 (*N* = 107)	99	99	95	2.74
	(92.52)	(92.52)	(88.79)	(0.54)
in 1993 (*N* = 163)	153	136	135	2.60
	(93.87)	(83.44)	(82.82)	(0.73)
in 1994 (*N* = 138)	113	127	124	2.64
	(81.88)	(92.03)	(89.86)	(0.64)
in 1995 (*N* = 181)	150	160	178	2.70
	(82.87)	(88.40)	(98.34)	(0.63)
in 1996 (*N* = 170)	129	152	167	2.64
	(75.88)	(89.41)	(98.24)	(0.66)
in 1997 (*N* = 134)	101	111	129	2.54
	(75.37)	(82.84)	(96.27)	(0.77)
in 1998 (*N* = 134)	104	110	132	2.58
	(77.61)	(82.09)	(98.51)	(0.75)
1999 (*N* = 106)	80	90	104	2.58
	(75.47)	(84.91)	(98.11)	(0.74)
Divorce				
No divorce (*N* = 14,349)	10,5815	11,302	13,083	2.46
	(75.37)	(78.76)	(91.17)	(0.82)
Divorce (*N* = 423)	352	374	389	2.64
	(83.22)	(88.42)	(91.96)	(0.67)
in 1991 (*N* = 45)	44	43	42	2.87
	(97.78)	(95.56)	(93.33)	(0.40)
in 1992 (*N* = 27)	24	23	25	2.67
	(88.89)	(85.19)	(92.59)	(0.73)
in 1993 (*N* = 50)	45	44	45	2.68
	(90.00)	(88.00)	(90.00)	(0.68)
in 1994 (*N* = 56)	43	52	45	2.50
	(76.79)	(92.86)	(80.36)	(0.71)
in 1995 (*N* = 67)	55	60	65	2.69
	(82.09)	(89.55)	(97.01)	(0.61)
in 1996 (*N* = 55)	39	46	53	2.51
	(70.91)	(83.64)	(96.36)	(0.74)
in 1997 (*N* = 54)	45	47	48	2.59
	(83.33)	(87.04)	(88.89)	(0.69)
in 1998 (*N* = 11)	10	10	10	2.73
	(90.91)	(90.91)	(90.91)	(0.65)
in 1999 (*N* = 58)	47	49	56	2.62
	(81.03)	(84.48)	(96.55)	(0.72)
Death of partner				
No death of partner (*N* = 14,335)	10,781	11,278	13,062	2.45
	(75.21)	(78.67)	(91.11)	(0.82)
Death of partner (*N* = 437)	386	398	410	2.73
	(88.33)	(91.08)	(93.82)	(0.60)
in 1991 (*N* = 41)	38	37	36	2.71
	(92.68)	(90.24)	(87.80)	(0.64)
in 1992 (*N* = 54)	50	47	48	2.69
	(92.59)	(87.04)	(88.89)	(0.70)
in 1993 (*N* = 56)	51	48	50	2.66
	(91.07)	(85.71)	(89.29)	(0.69)
in 1994 (*N* = 70)	58	68	65	2.73
	(82.86)	(97.14)	(92.86)	(0.54)
in 1995 (*N* = 57)	50	51	57	2.77
	(87.72)	(89.47)	(100.00)	(0.54)
in 1996 (*N* = 39)	33	35	35	2.64
	(84.62)	(89.74)	(89.74)	(0.67)
in 1997 (*N* = 41)	34	38	40	2.73
	(82.93)	(92.68)	(97.56)	(0.60)
in 1998 (*N* = 39)	37	37	39	2.90
	(94.87)	(94.87)	(100.00)	(0.38)
in 1999 (*N* = 40)	35	37	40	2.80
	(87.50)	(92.50)	(100.00)	(0.56)

*Note*. Percentages and standard deviations are in parenthesis.

**Table 3:** Sample characteristics in individuals who did and did not experience the respective loss between 1991 and 1999 (*N* = 14,772).

	Separation		Divorce		Death of partner	
	No (*N* = 13,537)	Yes (*N* = 1,235)	No (*N* = 14,349)	Yes (*N* = 423)	No (*N* = 14,335)	Yes (*N* = 437)
Female gender						
N	6,969	666	7,399	236	7,315	320
%	51.48	53.93	51.56	55.79	51.03	73.23
Male gender						
N	6,568	569	6,950	187	7,020	117
%	48.52	46.07	48.44	44.21	48.97	26.77
Age						
Grand-mean	44.71	32.89	43.89	35.93	43.00	62.72
SD	17.13	9.32	17.07	8.00	16.64	13.56
Past-loss ^1^						
N	289	94	178	8	191	1
%	2.13	7.61	1.24	1.89	1.33	0.23
Repeated loss ^2^						
N	-	167	-	14	-	3
%	-	13.52	-	3.31	-	0.69
Internal control beliefs						
Grand-mean	2.96	3.01	2.96	2.98	2.96	2.93
SD	0.52	0.49	0.52	0.48	0.51	0.62
External control beliefs						
Grand-mean	2.33	2.27	2.33	2.27	2.32	2.59
SD	0.62	0.60	0.62	0.61	0.61	0.63
Total control beliefs						
Grand-mean	2.77	2.84	2.78	2.82	2.79	2.60
SD	0.47	0.46	0.47	0.46	0.47	0.49

*Note*.

^1^ Individuals who indicated to have experienced the respective loss prior to 1991 (between 1984, the first wave of the SOEP, and 1990)

^2^ Individuals who indicated to have experienced the respective loss more than once between 1991 and 1999.

### Differences in perceived control between individuals with and without the respective loss

Differences in perceived control between individuals who did (*N* = 1,235) and did not (*N* = 13,537) separate from a partner between 1991 and 1999 are presented in Table 4. There were no selection effects, indicating that individuals who provided perceived control data and separated from a partner afterwards did not differ in their perceived control from individuals without this loss. However, a positive post-loss difference effect on internal control beliefs (*b =* 0.14) indicated that individuals who separated from a partner and provided perceived control data afterwards had higher internal control beliefs compared to individuals without this loss. Taken together, individuals who separated from a partner did not differ in their perceived control before but had higher internal control beliefs after this experience compared to individuals without this loss.

**Table 4:** Differences in perceived control between individuals who did (*N* = 1,235) and did not (*N* = 13,537) separate from a partner between 1991 and 1999 (*N* = 14,772).

	Internal control beliefs				External control beliefs				Total control beliefs			
Fixed effects	b	95% CI		p	b	95% CI		p	b	95% CI		p
Intercept	-0.01	-0.03	0.01	.235	-0.06	-0.08	-0.04	< .001	0.05	0.03	0.07	< .001
Gender	0.16	0.14	0.19	< .001	-0.19	-0.21	-0.18	< .001	0.23	0.21	0.25	< .001
Age	0.00	-0.01	0.02	.808	0.13	0.11	0.14	< .001	-0.10	-0.12	-0.09	< .001
Age^2^	0.01	0.00	0.01	.013	0.04	0.03	0.04	< .001	-0.03	-0.03	-0.02	< .001
Age^3^	0.00	-0.01	0.00	< .001	-0.01	-0.01	-0.01	< .001	0.01	0.00	0.01	< .001
Testing	0.01	0.00	0.02	.044	-0.06	-0.07	-0.05	< .001	0.05	0.04	0.06	< .001
Past-loss	0.07	-0.01	0.15	.088	-0.07	-0.14	0.01	.068	0.09	0.01	0.16	.024
Selection	0.05	-0.02	0.11	.147	-0.03	-0.09	0.03	.281	0.05	-0.01	0.11	.114
Post-loss difference	0.14	0.08	0.20	< .001	0.05	-0.01	0.11	.109	0.02	-0.04	0.07	.616
Random effects	Var.	95% CI			Var.	95% CI			Var.	95% CI		
Household (intercept)	0.31	0.29	0.33		0.46	0.44	0.48		0.43	0.41	0.46	
Person (intercept)	0.16	0.15	0.17		0.13	0.12	0.13		0.16	0.15	0.17	
Person (residual)	0.54	0.53	0.55		0.37	0.37	0.38		0.38	0.37	0.38	

*Note*. b = beta-coefficient from multilevel mixed-effect models; CI = confidence interval; p = p-value.

Differences in perceived control between individuals who did (*N* = 423) and did not (*N* = 14,349) get divorced between 1991 and 1999 are shown in Table 5. There were no selection and post-loss difference effects, indicating that individuals who got divorced did not differ in their perceived control from individuals without this loss (neither before nor after this experience).

**Table 5:** Differences in perceived control between individuals who did (*N* = 423) and did not (*N* = 14,349) get divorced between 1991 and 1999 (*N* = 14,772).

	Internal control beliefs				External control beliefs				Total control beliefs			
Fixed effects	b	95% CI		p	b	95% CI		p	b	95% CI		p
Intercept	0.00	-0.02	0.02	.775	-0.06	-0.08	-0.04	< .001	0.05	0.03	0.08	< .001
Gender	0.16	0.14	0.18	< .001	-0.19	-0.21	-0.18	< .001	0.23	0.21	0.25	< .001
Age	0.00	-0.02	0.01	.661	0.13	0.11	0.14	< .001	-0.11	-0.12	-0.09	< .001
Age^2^	0.01	0.00	0.01	.031	0.04	0.03	0.04	< .001	-0.03	-0.03	-0.02	< .001
Age^3^	0.00	-0.01	0.00	< .001	-0.01	-0.01	-0.01	< .001	0.01	0.00	0.01	< .001
Testing	0.01	0.00	0.02	.019	-0.06	-0.07	-0.05	< .001	0.05	0.04	0.06	< .001
Past-loss	0.14	0.03	0.25	.014	-0.06	-0.16	0.05	.278	0.11	0.00	0.22	.043
Selection	0.01	-0.09	0.12	.775	0.03	-0.07	0.12	.561	-0.02	-0.12	0.08	.680
Post-loss difference	0.02	-0.08	0.12	.634	0.00	-0.09	0.09	.965	0.01	-0.09	0.10	.873
Random effects	Var.	95% CI			Var.	95% CI			Var.	95% CI		
Household (intercept)	0.31	0.29	0.33		0.46	0.44	0.48		0.43	0.41	0.46	
Person (intercept)	0.16	0.15	0.17		0.12	0.12	0.13		0.16	0.15	0.17	
Person (residual)	0.54	0.53	0.55		0.37	0.37	0.38		0.38	0.37	0.38	

*Note*. b = beta-coefficient from multilevel mixed-effect models; CI = confidence interval; p = p-value.

Differences in perceived control between individuals who did (*N* = 437) and did not (*N* = 14,335) experience the death of a partner between 1991 and 1999 are presented in Table 6. No selection effects were found, indicating that individuals who provided perceived control data and experienced the death of a partner afterwards did not differ in their perceived control from individuals without this loss. However, a positive post-loss difference effect on internal control beliefs (*b =* 0.17) and external control beliefs (*b =* 0.15) indicated that individuals who experienced the death of a partner and provided perceived control data afterwards had higher internal control beliefs and higher external control beliefs compared to individuals without this loss. Taken together, individuals whose partner died did not differ in their perceived control before but had higher internal and external control beliefs after this experience compared to individuals without this loss.

**Table 6:** Differences in perceived control between individuals who did (*N* = 437) and did not (*N* = 14,335) experience the death of a partner between 1991 and 1999 (*N* = 14,772).

	Internal control beliefs				External control beliefs				Total control beliefs			
Fixed effects	b	95% CI		p	b	95% CI		p	b	95% CI		p
Intercept	0.00	-0.02	0.02	.932	-0.06	-0.08	-0.04	< .001	0.05	0.03	0.08	< .001
Gender	0.17	0.14	0.19	< .001	-0.19	-0.21	-0.17	< .001	0.23	0.21	0.25	< .001
Age	-0.01	-0.02	0.01	.335	0.12	0.11	0.14	< .001	-0.11	-0.12	-0.09	< .001
Age^2^	0.00	0.00	0.01	.144	0.03	0.03	0.04	< .001	-0.03	-0.03	-0.02	< .001
Age^3^	0.00	-0.01	0.00	.001	-0.01	-0.01	-0.01	< .001	0.01	0.00	0.01	< .001
Testing	0.01	0.00	0.02	.027	-0.06	-0.07	-0.05	< .001	0.05	0.04	0.06	< .001
Past-loss	0.14	0.03	0.26	.015	0.08	-0.03	0.19	.167	-0.01	-0.12	0.11	.894
Selection	-0.02	-0.11	0.07	.666	0.04	-0.04	0.13	.287	-0.04	-0.13	0.04	.323
Post-loss difference	0.17	0.07	0.26	.001	0.15	0.06	0.24	.001	-0.05	-0.14	0.04	.268
Random effects	Var.	95% CI			Var.	95% CI			Var.	95% CI		
Household (intercept)	0.31	0.29	0.33		0.46	0.44	0.48		0.43	0.41	0.46	
Person (intercept)	0.16	0.15	0.17		0.12	0.12	0.13		0.16	0.15	0.17	
Person (residual)	0.54	0.53	0.55		0.37	0.37	0.38		0.38	0.37	0.38	

*Note*. b = beta-coefficient from multilevel mixed-effect models; CI = confidence interval; p = p-value.

#### Interactions with gender

Testing interactions with gender revealed no significant results, indicating that the examined selection and post-loss difference effects did not differ between women and men.

#### Interactions with age

Testing interactions with age revealed no significant results for separation and divorce. That is, the examined selection and post-loss difference effects for separation and divorce did not differ between younger and older individuals. For the death of a partner, the post-loss difference effect on external control beliefs varied by age (*b =* -0.12; 95% CI: -0.19, 0.06; *p <* .001). To assess this interaction in greater detail, we conducted a grand-mean split of the dimensional age variable (*M* = 43.65; *SD =* 16.92 years) and distinguished between younger (< = 43 years) and older (>43 years) individuals. Separate models in both groups revealed that only younger (*b =* 0.64; 95% CI: 0.36, 0.93; *p <* .001) but not older individuals had higher external control beliefs after the death of a partner compared to same-aged individuals without this loss. Moreover, the post-loss difference effect on total control beliefs varied by age (*b =* 0.07; 95% CI: 0.01, 0.14; *p =* .032). Decomposing this interaction revealed that only younger (*b =* -0.46; 95% CI: -0.74, -0.17; *p =* .002) but not older individuals had lower total control beliefs after the death of a partner compared to same-aged individuals without this loss.

### Changes in perceived control around the respective loss

**Fig 1:**
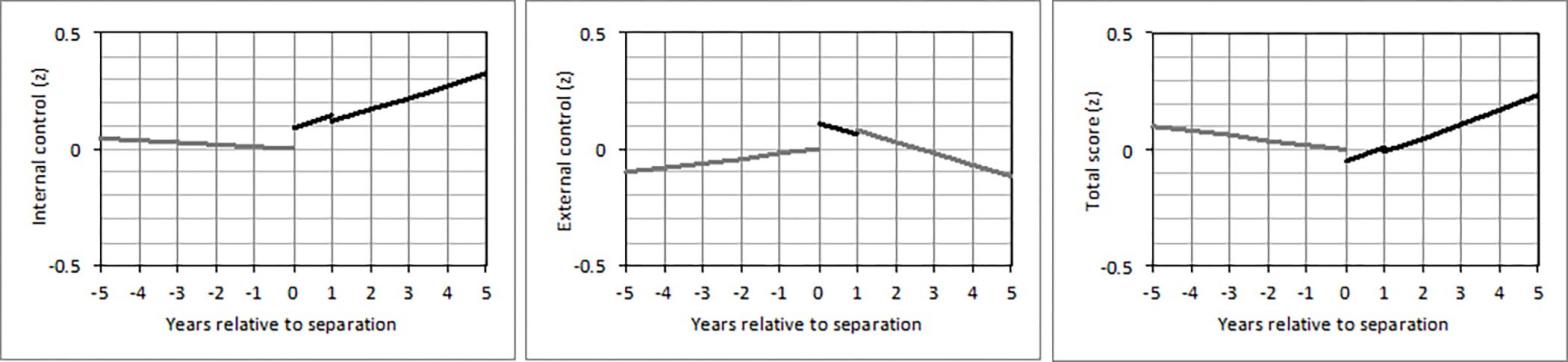
Changes in (a) internal, (b) external, and (c) total control beliefs from five years before until five years after separation from a partner in individuals who experienced this loss between 1991 and 1999 (*N* = 1,235). *Note*. The first line indicates changes of perceived control in the five years before separation from a partner. It is based on the anticipation effect multiplied by the time (in years) until this loss. The second line indicates changes of perceived control in the first year after separation from a partner. It is based on the socialization effect multiplied by the time after this loss and the short-term effect. The third line indicates changes in perceived control more than one year after separation from a partner. It is based on the socialization effect multiplied by the time after this loss and the long-term effect. A black line indicates that any of the effects during the respective time frame (second line: socialization effect and/or short-term effect; third line: socialization effect and/or long-term effect) reached statistical significance (*p* < .05).

Changes in perceived control around separation (*N =* 1,235) are shown in Table 7. Significant effects are visualized in Fig 1. A positive short-term effect on external control beliefs (*b =* 0.11) indicated that external control beliefs were higher in the first year of being separated versus all other years. Furthermore, a positive socialization effect on internal control beliefs (*b =* 0.05 per year) and total control beliefs (*b =* 0.06 per year) indicated that internal and total control beliefs increased gradually in the years after participants had separated from a partner. Taken together, individuals who separated from a partner had higher external control beliefs in the first year of being separated but increased in their internal and total control beliefs in the following years.

**Table 7:** Changes in perceived control before and after separation from a partner in individuals who experienced this loss between 1991 and 1999 (*N* = 1,235).

	Internal control beliefs				External control beliefs				Total control beliefs			
Fixed effects	b	95% CI		p	b	95% CI		p	b	95% CI		p
Intercept	-0.13	-0.24	-0.02	.018	-0.07	-0.18	0.04	.216	-0.01	-0.12	0.11	.911
Gender	0.11	0.03	0.19	.006	-0.25	-0.33	-0.17	< .001	0.25	0.17	0.33	< .001
Age	-0.11	-0.19	-0.02	.012	0.18	0.08	0.27	< .001	-0.19	-0.29	-0.10	< .001
Age^2^	0.04	0.01	0.07	.015	0.05	0.02	0.09	.004	-0.02	-0.06	0.01	.252
Age^3^	0.02	0.01	0.04	.009	-0.02	-0.04	0.00	.022	0.03	0.01	0.05	.001
Testing	-0.02	-0.06	0.01	.226	-0.05	-0.08	-0.01	.008	0.03	-0.01	0.06	.096
Past-loss	0.04	-0.12	0.19	.641	-0.12	-0.28	0.04	.153	0.11	-0.06	0.27	.197
Anticipation	-0.01	-0.05	0.02	.564	0.02	-0.02	0.05	.347	-0.02	-0.05	0.02	.268
Socialization	0.05	0.00	0.11	.038	-0.05	-0.10	0.00	.053	0.06	0.01	0.11	.014
Short-term	0.09	-0.02	0.20	.098	0.11	0.02	0.21	.022	-0.05	-0.15	0.05	.305
Long-term	0.07	-0.09	0.22	.399	0.13	-0.01	0.27	.078	-0.07	-0.21	0.08	.355
Random effects	Var.	95% CI			Var.	95% CI			Var.	95% CI		
Household (intercept)	0.19	0.13	0.26		0.37	0.30	0.45		0.37	0.30	0.45	
Person (intercept)	0.16	0.11	0.24		0.15	0.10	0.21		0.16	0.11	0.22	
Person (residual)	0.52	0.49	0.56		0.39	0.37	0.42		0.40	0.37	0.42	

*Note*. b = beta-coefficient from multilevel mixed-effect models; CI = confidence interval; p = p-value.

Changes in perceived control around divorce (*N* = 423) are shown in Table 8. Getting divorced was unrelated to changes of perceived control in the surrounding years (all p-values>.05).

**Table 8:** Changes in perceived control before and after divorce in individuals who experienced this loss between 1991 and 1999 (*N* = 423).

	Internal control beliefs				External control beliefs				Total control beliefs			
Fixed effects	b	95% CI		p	b	95% CI		p	b	95% CI		p
Intercept	-0.01	-0.19	0.17	.902	0.02	-0.17	0.21	.821	-0.02	-0.21	0.17	.833
Gender	0.17	0.02	0.31	.022	-0.27	-0.41	-0.13	< .001	0.30	0.15	0.44	< .001
Age	-0.08	-0.24	0.09	.362	0.27	0.08	0.45	.004	-0.26	-0.44	-0.08	.005
Age^2^	0.05	-0.04	0.14	.293	-0.02	-0.12	0.09	.768	0.03	-0.07	0.14	.503
Age^3^	0.06	-0.01	0.12	.078	-0.10	-0.17	-0.04	.003	0.11	0.04	0.18	.001
Testing	-0.01	-0.07	0.05	.739	-0.06	-0.12	0.00	.055	0.04	-0.02	0.10	.174
Past-loss	-0.06	-0.57	0.44	.806	0.16	-0.41	0.74	.578	-0.16	-0.73	0.41	.587
Anticipation	0.00	-0.06	0.05	.893	0.02	-0.04	0.08	.466	-0.02	-0.07	0.04	.589
Socialization	0.02	-0.07	0.10	.686	0.03	-0.05	0.11	.459	-0.02	-0.10	0.07	.682
Short-term	0.02	-0.15	0.20	.804	-0.10	-0.26	0.07	.257	0.09	-0.08	0.25	.304
Long-term	-0.05	-0.31	0.21	.711	-0.06	-0.30	0.19	.659	0.02	-0.23	0.26	.888
Random effects	Var.	95% CI			Var.	95% CI			Var.	95% CI		
Household (intercept)	0.03	0.00	9.92		0.41	0.29	0.57		0.35	0.22	0.54	
Person (intercept)	0.32	0.18	0.57		0.11	0.04	0.29		0.17	0.08	0.36	
Person (residual)	0.50	0.45	0.55		0.42	0.38	0.47		0.42	0.38	0.47	

*Note*. b = beta-coefficient from multilevel mixed-effect models; CI = confidence interval; p = p-value.

**Fig 2:**
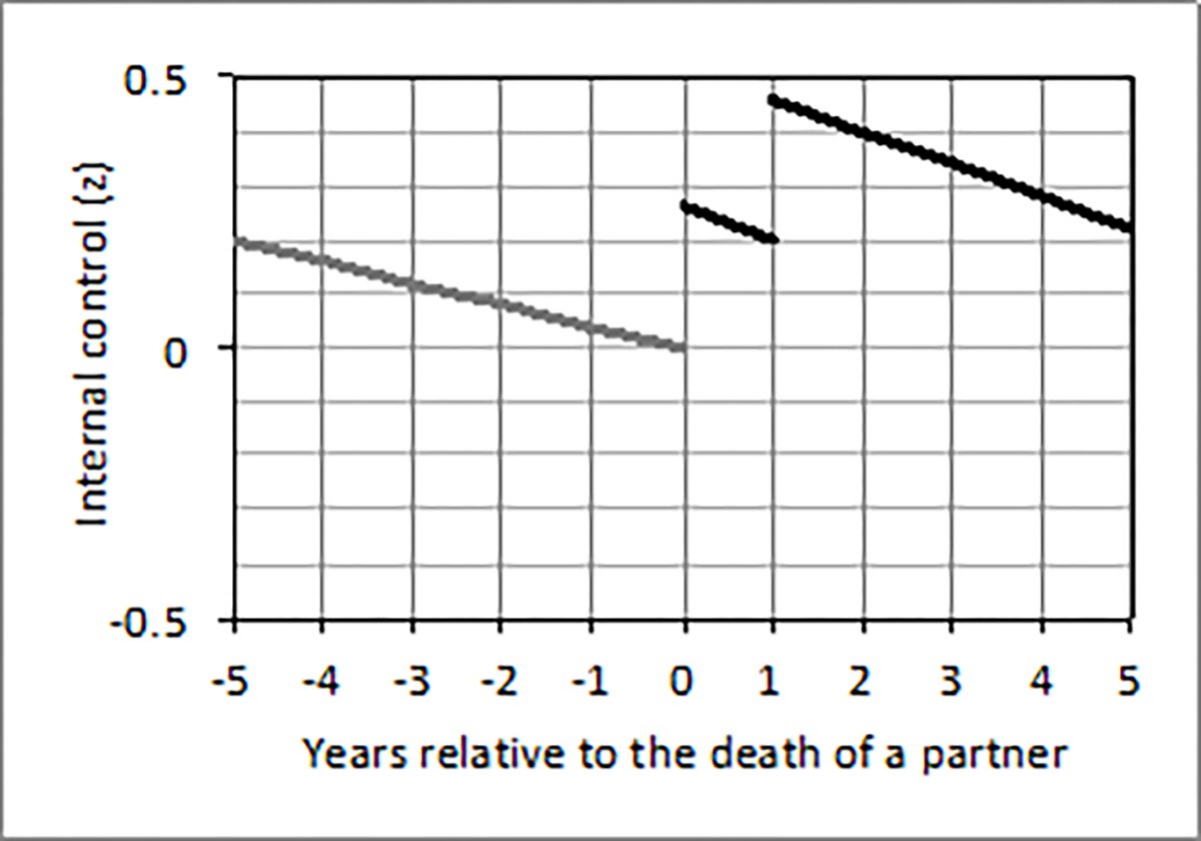
Changes of internal control beliefs from five years before until five years after the death of a partner in individuals who experienced this loss between 1991 and 1999 (*N* = 437). *Note*. The first line indicates changes of perceived control in the five years before the death of a partner. It is based on the anticipation effect multiplied by the time (in years) until this loss. The second line indicates changes of perceived control in the first year after the death of a partner. It is based on the socialization effect multiplied by the time after this loss and the short-term effect. The third line indicates changes in perceived control more than one year after the death of a partner. It is based on the socialization effect multiplied by the time after this loss and the long-term effect. The second line and third line are marked in black because the short-term (second line) and long-term (third line) post-loss effect reached statistical significance (*p* < .05).

Changes in perceived control around the death of a partner (*N* = 437) are shown in Table 9. A positive short-term (*b =* 0.26) and long-term (*b =* 0.52) effect on internal control beliefs indicated that internal control beliefs were higher in and especially beyond the first year after this experience versus before (Fig 2).

**Table 9:** Changes in perceived control before and after the death of a partner in individuals who experienced this loss between 1991 and 1999 (*N* = 437).

	Internal control beliefs				External control beliefs				Total control beliefs			
Fixed effects	b	95% CI		p	b	95% CI		p	b	95% CI		p
Intercept	-0.21	-0.46	0.03	.090	0.34	0.14	0.55	.001	-0.37	-0.59	-0.16	.001
Gender	0.30	0.09	0.51	.006	-0.27	-0.45	-0.09	.004	0.33	0.14	0.52	.001
Age	0.05	-0.11	0.21	.558	0.06	-0.08	0.20	.394	-0.03	-0.17	0.12	.700
Age^2^	0.04	-0.07	0.16	.459	0.00	-0.10	0.09	.931	0.02	-0.08	0.12	.719
Age^3^	-0.02	-0.04	0.00	.068	0.00	-0.02	0.02	.921	-0.01	-0.03	0.01	.462
Testing	0.04	-0.03	0.11	.275	-0.06	-0.12	-0.01	.026	0.07	0.01	0.13	.015
Past-loss	0.98	-1.34	3.31	.407	1.63	-0.34	3.60	.104	-0.95	-2.99	1.08	.358
Anticipation	-0.04	-0.11	0.03	.245	0.03	-0.03	0.08	.296	-0.04	-0.10	0.01	.128
Socialization	-0.06	-0.16	0.04	.234	0.01	-0.07	0.09	.742	-0.04	-0.12	0.05	.392
Short-term	0.26	0.05	0.47	.015	0.05	-0.11	0.21	.535	0.06	-0.11	0.23	.484
Long-term	0.52	0.23	0.82	< .001	-0.01	-0.24	0.22	.943	0.22	-0.02	0.46	.070
Random effects	Var.	95% CI			Var.	95% CI			Var.	95% CI		
Household (intercept)	0.30	0.04	2.46		0.57	0.48	0.68		0.61	0.52	0.73	
Person (intercept)	0.36	0.06	2.11		0.00	0.00	0.00		0.00	0.00	0.00	
Person (residual)	0.73	0.66	0.80		0.43	0.39	0.47		0.46	0.41	0.50	

*Note*. b = beta-coefficient from multilevel mixed-effect models; CI = confidence interval; p = p-value.

To account for individual differences in mean-level changes of perceived control around the respective loss, we repeated the analyses and included random effects for the anticipation, socialization, short-term, and long-term post-loss variable in the respective model. These analyses revealed highly similar results and none of the fixed effects reported above changed. In other words, our findings on mean-level changes were still seen when taking individual differences in these effects into account.

#### Interactions with gender

**Fig 3:**
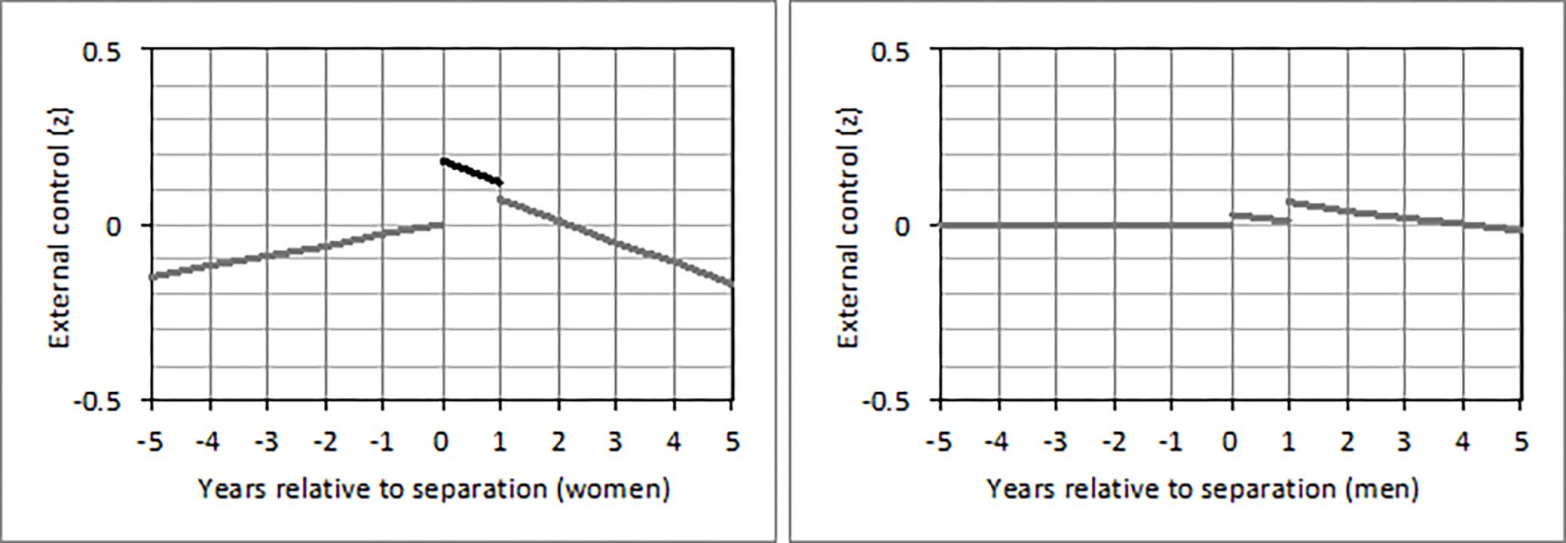
Changes of external control beliefs from five years before until five years after separation from a partner in (a) women and (b) men who experienced this loss between 1991 and 1999. *Note*. A detailed description of the figure can be found in Fig 1. The second line in (a) is marked in black because the short-term effect reached statistical significance (*p* < .05) only in women but not in men.

In individuals who separated from a partner, the short-term effect on external control beliefs differed between women and men (*b* = -0.16; 95% CI: -0.31, -0.02; *p* = .024). Decomposing this interaction revealed that only women (*b* = 0.18; 95% CI: 0.04, 0.31; *p* = .009) but not men had higher external control beliefs in the first year after breaking up versus all other years around this experience (Fig 3). No interactions with gender were found for divorce and the death of a partner.

#### Interactions with age

**Fig 4:**
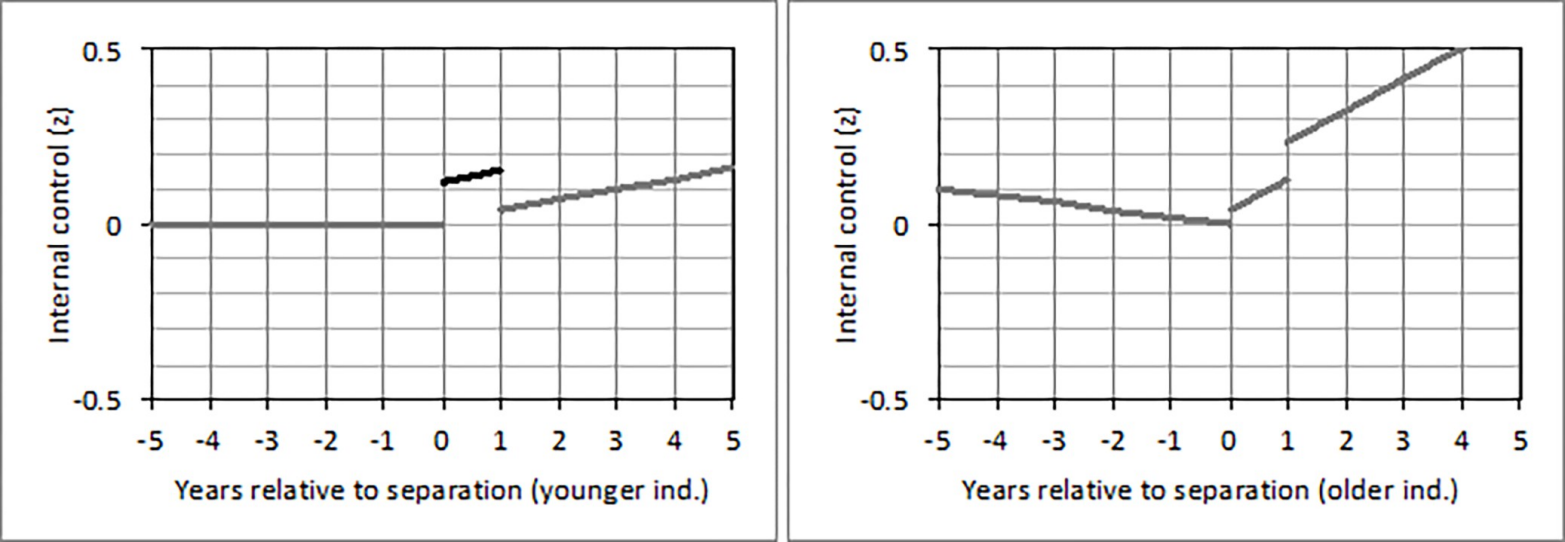
Changes of internal control beliefs from five years before until five years after separation from a partner in (a) younger and (b) older individuals who experienced this loss between 1991 and 1999. *Note*. A detailed description of the figure can be found in Fig 1. The second line in (a) is marked in black because the short-term effect reached statistical significance (*p* < .05) only in younger but not in older individuals.

In individuals who separated from a partner, the short-term effect on internal control beliefs varied by age (*b* = -0.10; 95% CI: -0.20, -0.01; *p* = .032). To assess this interaction in greater detail, we conducted a grand-mean split of the dimensional age variable (*M* = 32.89; *SD* = 9.32 years) and distinguished between younger (< = 32 years) and older (>32 years) individuals. Only younger (*b* = 0.12; 95% CI: -0.01, 0.26; *p* = .072) but not older individuals tended to have higher internal control beliefs in the first year of being separated versus all other years (Fig 4). Though, this effect was only marginally significant. In individuals who got divorced, no interactions with age were found.

**Fig 5:**
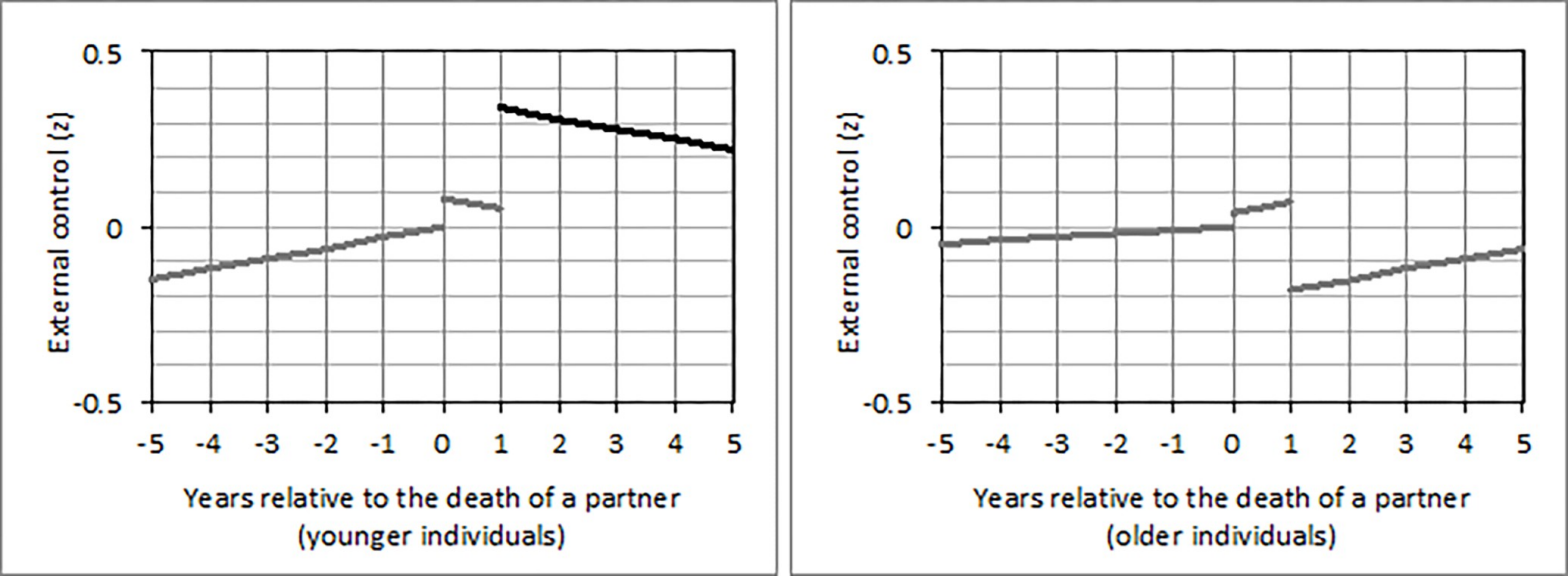
Changes of external control beliefs from five years before until five years after the death of a partner in (a) younger and (b) older individuals who experienced this loss between 1991 and 1999. *Note*. A detailed description of the figure can be found in Fig 2. The third line in (a) is marked in black because the long-term effect reached statistical significance (*p* < .05) only in younger but not in older individuals.

**Fig 6:**
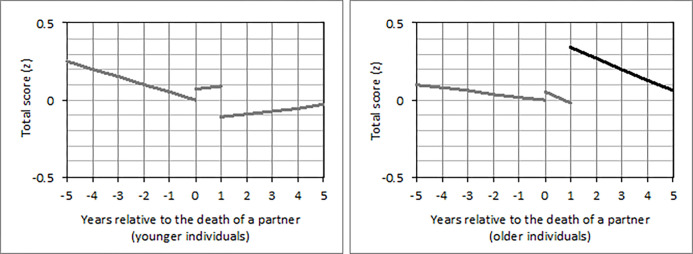
Changes of total control beliefs from five years before until five years after the death of a partner in (a) younger and (b) older individuals who experienced this loss between 1991 and 1999. *Note*. A detailed description of the figure can be found in Fig 2. The third line in (b) is marked in black because the long-term effect reached statistical significance (*p* < .05) only in older but not in younger individuals.

In individuals whose partner died, the long-term effect on external control beliefs varied by age (*b =* -0.14; 95% CI: -0.23, -0.05; *p =* .003). To assess this interaction in greater detail, we conducted a grand-mean split of the dimensional age variable (*M* = 62.72; *SD =* 13.56 years) and distinguished between younger (< = 62 years) and older (>62 years) individuals. These models revealed that only younger (*b =* 0.37; 95% CI: 0.01, 0.73; *p =* .046) but not older individuals had higher external control beliefs more than one year after their partner had died versus before (Fig 5). Moreover, the long-term effect on total control beliefs varied by age (*b =* 0.12; 95% CI: 0.03, 0.22; *p =* .012). That is, only older (*b =* 0.41; 95% CI: 0.11, 0.72; *p =* .009) but not younger individuals had higher total control beliefs more than one year after their partner had died versus before (Fig 6).

## Discussion

In this study, we used data from a nationally representative household panel study from Germany to examine the role of separation, divorce, and the death of a partner for perceived control. In the following sections, we will first relate our findings to our theoretical rationale and hypotheses and then discuss the main implications of our study.

### Differences in perceived control between individuals with and without the respective loss

#### Selection effects

Because no selection effects were found, neither our hypothesis that perceived control would be lower (H1) nor our hypothesis that perceived control would be higher (H2) in individuals who will versus will not experience relationship losses was supported.

Initially, we argued that individuals with higher perceived control might feel more capable to actively shape and improve their relationship and thus be less likely to break up [26]. At the same time, they might feel more capable to manage their life without a partner and thus be more likely to end their relationship in times of trouble [45, 46]. One could speculate whether both assumptions are true and counteract each other, resulting into non-significant effects.

#### Post-loss differences

Regarding post-loss differences, we found that individuals who separated from a partner and individuals who whose partner died had higher internal control beliefs after this experience compared to individuals without this loss. These results confirm our stress-related growth hypothesis that perceived control would be higher in individuals with versus without relationship losses in the past (H5). Moreover, these findings are consistent with cross-sectional evidence that perceived control was higher in single and divorced versus never married, married, and widowed individuals [45, 46]. After losing their partner, people might often realize to be able to deal with life without a partner and gain autonomy and independency [45, 46], leading to higher internal control beliefs. However, we also found that individuals had higher external control beliefs after their partner had died compared to individuals without this loss. This speaks in favor of our learned helplessness hypothesis that perceived control would be lower in individuals with versus without relationship losses in the past (H3). Being confronted with death might sharpen peoples’ awareness that life (e.g., health and ageing) can only partly be controlled by one’s own behavior, leading to higher external control beliefs in the following years. No post-loss differences in perceived control were found for divorce. That is, consistent with our set-point hypothesis (H4), individuals who got divorced did not differ in their perceived control after this experience from individuals without this loss. Taken together, our findings on post-loss differences suggest that (a) internal control beliefs increase after separation and (b) both internal and external control beliefs increase after the death of a partner. No effects, however, were found for divorce.

### Changes in perceived control around the respective loss

#### Anticipation effects

There were no anticipation effects, indicating that perceived control only changed after but not before relationship losses. These findings suggest that especially psychological challenges in the aftermath of such experiences might trigger changes in perceived control (e.g., disengaging from one’s ex-partner and adjusting to single life).

#### Short-term effects

We found that individuals who separated from a partner had higher external control beliefs in (but not after) the first year of being separated versus all other years. In line with Motivational Theory of Lifespan Development [1], Set-Point Theory [33], and Theory of Learned Helplessness [44], this result speaks in favor of the idea that perceived control might be lower in the first year after relationship losses (H6). Besides, this finding contradicts our learned helplessness hypothesis (H8) and stress-related growth hypothesis (H10) but supports our set-point hypothesis that perceived control would bounce back to its set-point after some time (H9). Moreover, this result is roughly consistent with previous evidence that women who broke up were less likely to change from low to high perceived control and more likely to change from high to low perceived control in the surrounding years [47]. However, this previous study did not distinguish between short- and long-term changes of perceived control in the years before and after separating from a partner. The fact that separation was followed by a transient but not enduring decrease of perceived control suggests that such a distinction is important. In line with Motivational Theory of Lifespan Development [1], people might switch to secondary control strategies after breaking up and tend to attribute their loss to external sources (e.g., their ex-partner, a love affair, or fate), which could explain this result.

Furthermore, individuals whose partner died had higher internal control beliefs in the first year after this experience. This result contradicts our hypothesis that perceived control would be lower in the first year after relationship losses (H6). As previously suggested [49], individuals often face substantial burden before their partner dies (e.g., due to caregiving). After losing their spouse, they might regain capabilities to shape their own life, which could explain this seemingly implausible finding.

No short-term effects were found for divorce. Thus, our hypothesis that perceived control would be lower in the first year after relationship losses (H6) was not supported for divorce.

Taken together, our study suggests that perceived control (a) decreases in the first year after separation but (b) increases in the first year after the death of a partner. However, no short-term effects were found for divorce.

#### Socialization and long-term effects

Our study revealed that individuals who separated from a partner increased gradually in their internal and total control beliefs in the following years. This result confirms our hypothesis that perceived control would increase gradually after relationship losses (H7). In line with Motivational Theory of Lifespan Development [1] and our finding that external control beliefs were higher in the first year of being separated, people might mainly use secondary control strategies in the first months after a breakup. This might help them to disengage from their ex-partner, overcome their loss, and shift to primary control strivings again, leading to a gradual increase of internal and total control beliefs. After breaking up, individuals might also realize to be able to deal with their life on their own and increase in autonomy and independency [45, 46]. Consistent with the idea of stress-related growth [41, 42], this might further boost perceived control.

With respect to long-term effects, our study revealed that individuals whose partner died had higher internal control beliefs more than one year after this experience versus before. This result contradicts our learned helplessness hypothesis (H8) and our set-point hypotheses (H9) but supports our stress-related growth hypothesis that perceived control would be higher more than one year after relationship losses compared to before (H10). After losing their spouse, individuals might not only regain capabilities to shape their own daily routines [49] but also recognize to be able to deal with life despite this tragic experience [43, 60], resulting into higher perceived control.

Finally, we found no long-term or socialization effects for divorce. Theoretically, these findings align with our set-point hypothesis that perceived control would not differ more than one year after relationship losses (H9). However, this hypothesis was based on the idea that perceived control would first decrease and then bounce back to its pre-loss levels. In our study, no evidence for such a trajectory was found.

Taken together, our study suggests that perceived control (a) increases gradually in the years after separation and (b) is higher in the years after the death of a partner than before. However, no socialization or long-term effects were found for divorce.

### Gender differences

Our study revealed that only women but not men had higher external control beliefs in the first year after separation compared to all other years. This finding confirms our hypothesis that women would experience a stronger decrease of perceived control in the first year after relationship losses than men (H11). Furthermore, this result is consistent with previous evidence that women but not men who broke up decreased in their perceived control [47]. In line with traditional gender roles, women might tend to more strongly focus on their relationship and family and thus feel to lose more control after relationship dissolution [46]. In addition, women might often face more serious family, financial, and work-related difficulties (e.g., due to single parenting) after separation and thus perceive the following months as particularly overwhelming and unmanageable, which could explain this result. For divorce and the death of a partner, our hypotheses on gender differences (H11) were not confirmed because no interactive effects were found.

### Age differences

We found that especially younger individuals had higher internal control beliefs in the first year after separation versus all other years. This result contradicts our hypothesis that younger (versus older) individuals would experience a stronger decrease of perceived control in the first year after relationship losses (H12). On average, younger individuals might have more energy and be more socially active. Therefore, they might feel more capable to overcome their loss, adjust to single life, and find a new partner after a breakup, which could explain this result.

In addition, especially younger individuals had higher external control beliefs and lower total control beliefs after the death of a partner compared to individuals without this loss. Moreover, especially younger individuals had higher external control beliefs, while especially older individuals had higher total control beliefs more than one year after their partner had died. These results support our hypothesis that younger individuals would experience a stronger decrease of perceived control after relationship losses (H12). However, in contrast to our hypothesis, our findings suggest that such age differences tend to manifest in the long (but not short) run. In young adulthood, the death of a partner constitutes a less prevalent and less normative event that (a) is often caused by unforeseen circumstances (e.g., an accident or a rare disease) and (b) may have dramatic consequences on affected individuals’ entire future life (e.g., because they must raise common children on their own) [36, 52]. This could explain more detrimental long-term effects among younger versus older individuals. For divorce, our hypotheses on age differences (H12) were not confirmed because no interactive effects were found.

## General discussion

Our study revealed that external control beliefs were higher in the first year after separation (compared to all other years around this loss). At the same time, perceived control increased gradually in the years after separation and was higher in the years after the death of a partner than before. Thus, our results largely confirm the idea that perceived control might increase after relationship losses (stress-related growth hypothesis) [41–43]. In contrast, the idea that perceived control might decrease after relationship losses received little support (learned helplessness hypothesis). The assumption that perceived control might first decrease and then bounce back to its pre-loss levels after relationship losses (set-point hypothesis) was partially confirmed: External control beliefs were lower in but not beyond the first year after separation. This finding is also consistent with Motivational Theory of Lifespan Development [1] and the idea that secondary control strategies and external control beliefs might be more pronounced in the first months after relationship losses.

Several aspects of our study are particularly noteworthy: First, our findings varied considerably for different types of relationship losses. For example, external control beliefs were higher in the first year after separation, whereas internal control beliefs were higher in the first year after the death of a partner. In all our models, divorce was unrelated to perceived control. That is, none of our hypotheses was confirmed for this type of loss. Individuals who undergo the judicial act of divorce might experience comparatively few changes in everyday life (e.g., because they already live separately from their partner), which could explain these results.

Second, changes in perceived control varied at different time points around the respective loss. There were no anticipation effects, which contradicts the idea that perceived control might change already prior to separation, divorce, and the death of a spouse. Internal control beliefs increased gradually after separation but suddenly after the death of a partner. Moreover, some changes seemed to be transient (e.g., the short-term effect on external control beliefs after separation) and others lasting (e.g., the long-term effect on internal control beliefs after the death of a partner). In line with previous research [29–31, 59, 61–63], these findings emphasize the importance to take time seriously and distinguish between continuous and discontinuous short- and long-term effects around major life experiences.

Third, our findings partially varied for internal and external control beliefs. For example, individuals whose partner died had higher internal control beliefs and—at the same time—higher external control beliefs after this experience compared to individuals without this loss. This highlights the importance to conceptualize internal and external control beliefs as two separate dimensions [4, 14, 15].

Fourth, our findings partially varied by gender and age. Women but not men had higher external control beliefs and particularly younger individuals had higher internal control beliefs after separating from a partner. Furthermore, the death of a partner had more detrimental effects on perceived control in younger versus older individuals. These findings highlight the impact of gender and age on loss-related changes in perceived control.

### Strengths and limitations

We used large-scaled longitudinal data from a nationally representative and heterogeneous sample from Germany (*N* = 14,772) covering the entire adult life span from young adulthood to old age. Relationship losses were yearly assessed, and perceived control was measured repeatedly across three consecutive years. This allowed us to model nuanced changes in perceived control around these losses, including gender and age effects. However, our study is not without limitations, which should be mentioned here.

First, to model short- and long-term changes in perceived control, we combined within- and between-person information. Second, perceived control was assessed with a relatively short scale of eight items. More comprehensive perceived control assessments might be more reliable and allow for more detailed analyses of domain-specific perceived control. Third, changes in perceived control around relationship losses might depend on additional factors related to the loss experience (e.g., predictability, perception, and subjective importance), the affected individual (e.g., attachment styles), the relationship (e.g., length and quality), and the socio-cultural environment more broadly (e.g., social support, culture, and religion). Additional research is necessary to assess the role of these factors in greater detail. Fourth, we used data from a representative sample from Germany, and the generalizability to other countries might be limited.

## Conclusions

Our findings suggest that relationship losses relate to a short-term decrease but long-term increase of perceived control. Strictly prospective-longitudinal studies are warranted to examine pure within-person changes of perceived control in the years around separation, divorce, the death of a partner, as well as other interpersonal loss experiences. Moreover, additional research is needed to zoom into people’s everyday life and examine the underlying mechanisms (e.g., changes in social roles and role demands as a partnered versus single person) that might explain changes in perceived control around such experiences.

## Supporting information

S1 Table Examples for the coding of the selection/post-loss difference, anticipation, socialization, short-term, and long-term variable. (DOCX)

S1 File Analytic codes. (DOCX)
