# Supplementary material for: Personality growth after relationship losses: Changes of perceived control in the years around separation, divorce, and the death of a partner
Source: PLoS One. 2022 Aug 3;17(8):e0268598. doi: 10.1371/journal.pone.0268598 (PMC9348722; doi:10.1371/journal.pone.0268598)
Supplement: S1 Table — (DOCX) [file pone.0268598.s001.docx]

Appendix 1: Table S1

*Examples for the coding of the selection/ post-loss difference, anticipation, socialization, short-term, and long-term variable*

|  | Assessment of perceived control  in 1994 (*N* = 11,167) | | | | | Assessment of perceived control  in 1995 (*N* = 11,676) | | | | | Assessment of perceived control  in 1996 (*N* = 13,472) | | | | |
| --- | --- | --- | --- | --- | --- | --- | --- | --- | --- | --- | --- | --- | --- | --- | --- |
| Respective loss | Select/ post-loss diff. | Ant | Soc | Short-term | Long-term | Select/ post-loss diff. | Ant | Soc | Short-term | Long-term | Select/ post-loss diff. | Ant | Soc | Short-term | Long-term |
| Not experienced | 0 | 0 | 0 | 0 | 0 | 0 | 0 | 0 | 0 | 0 | 0 | 0 | 0 | 0 | 0 |
| Experienced |  |  |  |  |  |  |  |  |  |  |  |  |  |  |  |
| in 1991 | 2 | 0 | 3 | 0 | 1 | 2 | 0 | 4 | 0 | 1 | 2 | 0 | 5 | 0 | 1 |
| in 1992 | 2 | 0 | 2 | 0 | 1 | 2 | 0 | 3 | 0 | 1 | 2 | 0 | 4 | 0 | 1 |
| in 1993 | 2 | 0 | 1 | 0 | 1 | 2 | 0 | 2 | 0 | 1 | 2 | 0 | 3 | 0 | 1 |
| in 1994 | 2 | 0 | 0 | 1 | 0 | 2 | 0 | 1 | 0 | 1 | 2 | 0 | 2 | 0 | 1 |
| in 1995 | 1 | -1 | 0 | 0 | 0 | 2 | 0 | 0 | 1 | 0 | 2 | 0 | 1 | 0 | 1 |
| in 1996 | 1 | -2 | 0 | 0 | 0 | 1 | -1 | 0 | 0 | 0 | 2 | 0 | 0 | 1 | 0 |
| in 1997 | 1 | -3 | 0 | 0 | 0 | 1 | -2 | 0 | 0 | 0 | 1 | -1 | 0 | 0 | 0 |
| in 1998 | 1 | -4 | 0 | 0 | 0 | 1 | -3 | 0 | 0 | 0 | 1 | -2 | 0 | 0 | 0 |
| in 1999 | 1 | -5 | 0 | 0 | 0 | 1 | -4 | 0 | 0 | 0 | 1 | -3 | 0 | 0 | 0 |

*Note.* Select/ post-loss diff. = selection and post-loss difference variable; Ant = anticipation variable; Soc = socialization variable; Short-term = short-term variable; Long-term = long-term variable. Examples are given for full years only. In the analyses, more fine-grained information was used. That is, the temporal association between the respective loss and the respective assessment of perceived control was coded in years and months. For example, in 1994, the selection/ post-loss difference variable was coded with 0 in individuals without the respective loss, with 1 in individuals who experienced the respective loss in the following years (i.e., in 1995, 1996, 1997, 1998, or 1999), and with 2 in individuals who already had experienced the respective loss (i.e., in 1991, 1992, 1993, or 1994).
