# Supplementary material for: Personality growth after relationship losses: Changes of perceived control in the years around separation, divorce, and the death of a partner
Source: PLoS One. 2022 Aug 3;17(8):e0268598. doi: 10.1371/journal.pone.0268598 (PMC9348722; doi:10.1371/journal.pone.0268598)
Supplement: S1 File — (DOCX) [file pone.0268598.s002.docx]

Appendix 2: Analytic codes

clear

clear matrix

clear mata

set maxvar 32000

version 14.2

set more off

cd "XXX"

*include information from the meta file ppfad

use persnr sex gebjahr ?hhnr ??hhnr psample using ppfad

label variable persnr `"person ID"'

label variable sex `"gender"'

label variable gebjahr `"year of birth"'

mvdecode persnr sex gebjahr ?hhnr ??hhnr, mv(-1 -2 -3 -4 -5 -6 -7 -8 -9)

*include information on perceived control in 1994, 1995, and 1996

merge persnr using kp, sort keep(kp7101 kp7102 kp7103 kp7104 kp7105 kp7106 kp7107 kp7108) nokeep

drop _merge

merge persnr using lp, sort keep(lp0801 lp0802 lp0803 lp0804 lp0805 lp0806 lp0807 lp0808) nokeep

drop _merge

merge persnr using mp, sort keep(mp1001 mp1002 mp1003 mp1004 mp1005 mp1006 mp1007 mp1008) nokeep

drop _merge

mvdecode kp71?? lp08?? mp10??, mv(-1 -2 -3 -4 -5 -6 -7 -8 -9)

*reverse perceived control items

for any kp7101 kp7102 kp7103 kp7104 kp7105 kp7106 kp7107 kp7108 lp0801 lp0802 lp0803 lp0804 lp0805 lp0806 lp0807 lp0808 mp1001 mp1002 mp1003 mp1004 mp1005 mp1006 mp1007 mp1008: tab X

for any kp7101 kp7102 kp7103 kp7104 kp7105 kp7106 kp7107 kp7108 lp0801 lp0802 lp0803 lp0804 lp0805 lp0806 lp0807 lp0808 mp1001 mp1002 mp1003 mp1004 mp1005 mp1006 mp1007 mp1008: replace X=5-X

for any kp7101 kp7102 kp7103 kp7104 kp7105 kp7106 kp7107 kp7108 lp0801 lp0802 lp0803 lp0804 lp0805 lp0806 lp0807 lp0808 mp1001 mp1002 mp1003 mp1004 mp1005 mp1006 mp1007 mp1008: tab X

*generate reversed items for external control

for any kp7102 kp7104 kp7105 kp7107 kp7108 lp0802 lp0804 lp0805 lp0807 lp0808 mp1002 mp1004 mp1005 mp1007 mp1008: gen Xr=5-X

*generate a variable for internal control in 1994, 1995, and 1996, respectively

egen internal94=rowmean(kp7101 kp7103 kp7106)

egen internal95=rowmean(lp0801 lp0803 lp0806)

egen internal96=rowmean(mp1001 mp1003 mp1006)

*generate a variable for external control in 1994, 1995, and 1996, respectively

egen external94=rowmean(kp7102 kp7104 kp7105 kp7107 kp7108)

egen external95=rowmean(lp0802 lp0804 lp0805 lp0807 lp0808)

egen external96=rowmean(mp1002 mp1004 mp1005 mp1007 mp1008)

*generate a variable for total control in 1994, 1995, and 1996, respectively

egen total94=rowmean(kp7101 kp7103 kp7106 kp7102r kp7104r kp7105r kp7107r kp7108r)

egen total95=rowmean(lp0801 lp0803 lp0806 lp0802r lp0804r lp0805r lp0807r lp0808r)

egen total96=rowmean(mp1001 mp1003 mp1006 mp1002r mp1004r mp1005r mp1007r mp1008r)

*recode gender (0=female, 1=male)

rename sex sex12

recode sex12 (1=1 male) (2=0 female), gen(sex)

drop sex12

label variable sex `"gender (0=f, 1=m)"'

*merge information from 1984 until 2004

*1984

merge persnr using ap, sort

drop _merge

*1985

merge persnr using bp, sort

drop _merge

*1986

merge persnr using cp, sort

drop _merge

*1987

merge persnr using dp, sort

drop _merge

*1988

merge persnr using ep, sort

drop _merge

*1989

merge persnr using fp, sort

drop _merge

*1990

merge persnr using gp, sort

drop _merge

*1991

merge persnr using hp, sort

drop _merge

*1992

merge persnr using ip, sort

drop _merge

*1993

merge persnr using jp, sort

drop _merge

*1994

merge persnr using kp, sort

drop _merge

*1995

merge persnr using lp, sort

drop _merge

*1996

merge persnr using mp, sort

drop _merge

*1997

merge persnr using np, sort

drop _merge

*1998

merge persnr using op, sort

drop _merge

*1999

merge persnr using pp, sort

drop _merge

*2000

merge persnr using qp, sort

drop _merge

*2001

merge persnr using rp, sort

drop _merge

*2002

merge persnr using sp, sort

drop _merge

*2003

merge persnr using tp, sort

drop _merge

*2004

merge persnr using up, sort

drop _merge

*mvdecode relationshiip losses

mvdecode bp80?? cp91?? dp93?? ep84?? fp103?? gp103?? hp103?? ip103?? jp103?? kp103?? lp103?? mp108?? np115?? op121?? pp133?? qp142??, mv(-1 -2 -3 -4 -5 -6 -7 -8 -9)

*mvdecode month of the respective interview

mvdecode bpmonin cpmonin dpmonin epmonin fpmonin gpmonin hpmonin ipmonin jpmonin kpmonin lpmonin mpmonin npmonin opmonin ppmonin qpmonin, mv(-1 -2 -3 -4 -5 -6 -7 -8 -9)

*generate variables that indicate the month of interview in 1994, 1995, and 1996, respectively

gen interview94=1994+kpmonin/12-1/12

gen interview95=1995+lpmonin/12-1/12

gen interview96=1996+mpmonin/12-1/12

*recode perceived control to missing when no information on the year and month of interview was available

for any 94 95 96: replace internalX=. if interviewX==.

for any 94 95 96: replace externalX=. if interviewX==.

for any 94 95 96: replace totalX=. if interviewX==.

*keep only individuals with information on perceived control in 1994, 1995, or 1996

keep if total94<. | total95<. | total96<.

save "XXXControl/control.dta", replace

//////////////////////////////////////////////////////////////

cd "XXXControl/"

use control, clear

*generate a variable that indicates the year and month of the respective loss

/*

*separation

*per year from 1984 to 1999

gen event99=.

replace event99=1 if (pp13310==1 & pp13312<.) | (qp14210==1 & qp14211<.)

gen event98=.

replace event98=1 if op12108<. | (pp13310==1 & pp13311<.)

gen event97=.

replace event97=1 if np11508<. | op12107<.

gen event96=.

replace event96=1 if mp10808<. | np11507<.

gen event95=.

replace event95=1 if lp10308<. | mp10807<.

gen event94=.

replace event94=1 if kp10308<. | lp10307<.

gen event93=.

replace event93=1 if jp10308<. | kp10307<.

gen event92=.

replace event92=1 if ip10308<. | jp10307<.

gen event91=.

replace event91=1 if hp10308<. | ip10307<.

gen event90=.

replace event90=1 if gp10308<. | hp10307<.

gen event89=.

replace event89=1 if fp10308<. | gp10307<.

gen event88=.

replace event88=1 if ep8408<. | fp10307<.

gen event87=.

replace event87=1 if dp9308<. | ep8407<.

gen event86=.

replace event86=1 if cp9108<. | dp9307<.

gen event85=.

replace event85=1 if bp8008<. | cp9107<.

gen event84=.

replace event84=1 if bp8007<.

*per month from 1991 to 1999

gen event_m99=pp13312

replace event_m99=qp14211 if pp13312==.

gen event_m98=op12108

replace event_m98=pp13311 if op12108==.

gen event_m97=np11508

replace event_m97=op12107 if np11508==.

gen event_m96=mp10808

replace event_m96=np11507 if mp10808==.

gen event_m95=lp10308

replace event_m95=mp10807 if lp10308==.

gen event_m94=kp10308

replace event_m94=lp10307 if kp10308==.

gen event_m93=jp10308

replace event_m93=kp10307 if jp10308==.

gen event_m92=ip10308

replace event_m92=jp10307 if ip10308==.

gen event_m91=hp10308

replace event_m91=ip10307 if hp10308==.

*/

*divorce

*per year from 1984 to 1999

gen event99=.

replace event99=1 if (pp13307==1 & pp13309<.) | (qp14207==1 & qp14208<.)

gen event98=.

replace event98=1 if op12106<. | (pp13301==1 & pp13308<.)

gen event97=.

replace event97=1 if np11506<. | op12105<.

gen event96=.

replace event96=1 if mp10806<. | np11505<.

gen event95=.

replace event95=1 if lp10306<. | mp10805<.

gen event94=.

replace event94=1 if kp10306<. | lp10305<.

gen event93=.

replace event93=1 if jp10306<. | kp10305<.

gen event92=.

replace event92=1 if ip10306<. | jp10305<.

gen event91=.

replace event91=1 if hp10306<. | ip10305<.

gen event90=.

replace event90=1 if gp10306<. | hp10305<.

gen event89=.

replace event89=1 if fp10306<. | gp10305<.

gen event88=.

replace event88=1 if ep8406<. | fp10305<.

gen event87=.

replace event87=1 if dp9306<. | ep8405<.

gen event86=.

replace event86=1 if cp9106<. | dp9305<.

gen event85=.

replace event85=1 if bp8006<. | cp9105<.

gen event84=.

replace event84=1 if bp8005<.

*per month from 1991 to 1999

gen event_m99=pp13309

replace event_m99=qp14208 if pp13309==.

gen event_m98=op12106

replace event_m98=pp13308 if op12106==.

gen event_m97=np11506

replace event_m97=op12105 if np11506==.

gen event_m96=mp10806

replace event_m96=np11505 if mp10806==.

gen event_m95=lp10306

replace event_m95=mp10805 if lp10306==.

gen event_m94=kp10306

replace event_m94=lp10305 if kp10306==.

gen event_m93=jp10306

replace event_m93=kp10305 if jp10306==.

gen event_m92=ip10306

replace event_m92=jp10305 if ip10306==.

gen event_m91=hp10306

replace event_m91=ip10305 if hp10306==.

/*

*death of partner

*per year from 1984 to 1999

gen event99=.

replace event99=1 if (pp13313==1 & pp13315<.) | (qp14213==1 & qp14214<.)

gen event98=.

replace event98=1 if op12110<. | (pp13313==1 & pp13314<.)

gen event97=.

replace event97=1 if np11510<. | op12109<.

gen event96=.

replace event96=1 if mp10810<. | np11509<.

gen event95=.

replace event95=1 if lp10310<. | mp10809<.

gen event94=.

replace event94=1 if kp10310<. | lp10309<.

gen event93=.

replace event93=1 if jp10310<. | kp10309<.

gen event92=.

replace event92=1 if ip10310<. | jp10309<.

gen event91=.

replace event91=1 if hp10310<. | ip10309<.

gen event90=.

replace event90=1 if gp10310<. | hp10309<.

gen event89=.

replace event89=1 if fp10310<. | gp10309<.

gen event88=.

replace event88=1 if ep8410<. | fp10309<.

gen event87=.

replace event87=1 if dp9310<. | ep8409<.

gen event86=.

replace event86=1 if cp9110<. | dp9309<.

gen event85=.

replace event85=1 if bp8010<. | cp9109<.

gen event84=.

replace event84=1 if bp8009<.

*per month from 1991 to 1999

gen event_m99=pp13315

replace event_m99=qp14214 if pp13315==.

gen event_m98=op12110

replace event_m98=pp13314 if op12110==.

gen event_m97=np11510

replace event_m97=op12109 if np11510==.

gen event_m96=mp10810

replace event_m96=np11509 if mp10810==.

gen event_m95=lp10310

replace event_m95=mp10809 if lp10310==.

gen event_m94=kp10310

replace event_m94=lp10309 if kp10310==.

gen event_m93=jp10310

replace event_m93=kp10309 if jp10310==.

gen event_m92=ip10310

replace event_m92=jp10309 if ip10310==.

gen event_m91=hp10310

replace event_m91=ip10309 if hp10310==.

*/

*add the year to the respective monthly variable

for any 91 92 93 94 95 96 97 98 99: replace event_mX=event_mX/12-1/12

for any 91 92 93 94 95 96 97 98 99: replace event_mX=1900+X+event_mX

*generate a variable that indicates the number of the same event between 1991 and 1999

gen numberevent=0

for any 91 92 93 94 95 96 97 98 99: replace numberevent=numberevent+1 if eventX==1

replace numberevent=. if numberevent==0

save control02, replace

//////////////////////////////////////////////////////////////

use control02, clear

*generate a variable that indicates whether the respective loss occurred before 1991

gen pastevent=0

for any 84 85 86 87 88 89 90: replace pastevent=1 if eventX==1

*generate a variable that indicates if and when the respective loss occurred between 1991 and 1999 -> in monthly increments

*note: when the respective loss occurred more than once, the first occurrence was considered

gen event_m=.

for any 99 98 97 96 95 94 93 92 91: replace event_m=event_mX if eventX==1

*generate a variable that indicates if and when the respective loss occurred between 1991 and 1999 -> in yearly increments only

capture drop event_y

gen event_y=.

for any 91 92 93 94 95 96 97 98 99: replace event_y=19X if event_m>=19X & event_m<19X+1

replace event_y=. if event_m==.

*generate a variable that distinguishes between individuals with and without the respective loss between 1991 and 1999

gen eventdi=0

replace eventdi=1 if event_m<.

*generate a variable that indicates the time span (in years and months) between the respective loss and the respective assessment of perceived control

for any 94 95 96: gen timeX=.

for any 94 95 96: replace timeX=interviewX-event_m

*generate a variable "age_event" that indicates the age at the time point of the respective loss

gen age_event=.

for any 91 92 93 94 95 96 97 98 99: replace age_event=19X-gebjahr if event_m>=19X & event_m<19X+1

*generate linear age variables

gen age94=1994-gebjahr

gen age95=1995-gebjahr

gen age96=1996-gebjahr

*generate a variable "testing" that indicates the number of previous assessments of perceived control in 1994, 1995, and 1996, respectively

gen test94=0

gen test95=0

replace test95=1 if total94<.

gen test96=0

replace test96=1 if total94<.

replace test96=2 if total94<. & total95<.

*generate a variable "household" that indicates the household number during the first assessment of perceived control

*mvdecode month of interview

mvdecode khhnr lhhnr mhhnr, mv(-1 -2 -3 -4 -5 -6 -7 -8 -9)

gen hh=khhnr

replace hh=lhhnr if total94==.

replace hh=mhhnr if total94==. & total95==.

save control03, replace

//////////////////////////////////////////////////////////////

use control03, clear

*mvdecode life satisfaction

mvdecode ap6801 bp9301 cp9601 dp9801 ep89 fp108 gp109 hp10901 ip10901 jp10901 kp10401 lp10401 mp11001 np11701 op12301 pp13501 qp14301 rp13501 sp13501 tp14201 up14501, mv(-1 -2 -3 -4 -5 -6 -7 -8 -9)

*rename life satisfaction items

rename ap6801 ls1984

rename bp9301 ls1985

rename cp9601 ls1986

rename dp9801 ls1987

rename ep89 ls1988

rename fp108 ls1989

rename gp109 ls1990

rename hp10901 ls1991

rename ip10901 ls1992

rename jp10901 ls1993

rename kp10401 ls1994

rename lp10401 ls1995

rename mp11001 ls1996

rename np11701 ls1997

rename op12301 ls1998

rename pp13501 ls1999

rename qp14301 ls2000

rename rp13501 ls2001

rename sp13501 ls2002

rename tp14201 ls2003

rename up14501 ls2004

*generate the difference between the life satisfaction score five years after the respective loss and the life satisfaction score in the last year before the respective loss

for any 1991 1992 1993 1994 1995 1996 1997 1998 1999: gen ls5_X=.

for any 1984 1985 1986 1987 1988 1989 1990 /*1991 1992 1993 1994 1995 1996 1997 1998 1999 2000 2001 2002 2003 2004 2005 2006 2007 2008 2009 2010 2011 2012 2013 2014 2015 2016*/: replace ls5_1991=ls1996-lsX if lsX<.

for any 1984 1985 1986 1987 1988 1989 1990 1991 /*1992 1993 1994 1995 1996 1997 1998 1999 2000 2001 2002 2003 2004 2005 2006 2007 2008 2009 2010 2011 2012 2013 2014 2015 2016*/: replace ls5_1992=ls1997-lsX if lsX<.

for any 1984 1985 1986 1987 1988 1989 1990 1991 1992 /*1993 1994 1995 1996 1997 1998 1999 2000 2001 2002 2003 2004 2005 2006 2007 2008 2009 2010 2011 2012 2013 2014 2015 2016*/: replace ls5_1993=ls1998-lsX if lsX<.

for any 1984 1985 1986 1987 1988 1989 1990 1991 1992 1993 /*1994 1995 1996 1997 1998 1999 2000 2001 2002 2003 2004 2005 2006 2007 2008 2009 2010 2011 2012 2013 2014 2015 2016*/: replace ls5_1994=ls1999-lsX if lsX<.

for any 1984 1985 1986 1987 1988 1989 1990 1991 1992 1993 1994 /*1995 1996 1997 1998 1999 2000 2001 2002 2003 2004 2005 2006 2007 2008 2009 2010 2011 2012 2013 2014 2015 2016*/: replace ls5_1995=ls2000-lsX if lsX<.

for any 1984 1985 1986 1987 1988 1989 1990 1991 1992 1993 1994 1995 /*1996 1997 1998 1999 2000 2001 2002 2003 2004 2005 2006 2007 2008 2009 2010 2011 2012 2013 2014 2015 2016*/: replace ls5_1996=ls2001-lsX if lsX<.

for any 1984 1985 1986 1987 1988 1989 1990 1991 1992 1993 1994 1995 1996 /*1997 1998 1999 2000 2001 2002 2003 2004 2005 2006 2007 2008 2009 2010 2011 2012 2013 2014 2015 2016*/: replace ls5_1997=ls2002-lsX if lsX<.

for any 1984 1985 1986 1987 1988 1989 1990 1991 1992 1993 1994 1995 1996 1997 /*1998 1999 2000 2001 2002 2003 2004 2005 2006 2007 2008 2009 2010 2011 2012 2013 2014 2015 2016*/: replace ls5_1998=ls2003-lsX if lsX<.

for any 1984 1985 1986 1987 1988 1989 1990 1991 1992 1993 1994 1995 1996 1997 1998 /*1999 2000 2001 2002 2003 2004 2005 2006 2007 2008 2009 2010 2011 2012 2013 2014 2015 2016*/: replace ls5_1999=ls2004-lsX if lsX<.

gen ls5=.

for any 1991 1992 1993 1994 1995 1996 1997 1998 1999: replace ls5=ls5_X if event_y==X

replace ls5=0 if event_y==.

save control03, replace

//////////////////////////////////////////////////////////////

use control03, clear

*reduce the data to all relevant variables

keep persnr event_m age* time* sex internal* external* total* test* pastevent eventdi numberevent hh ls5 event_y

*reshape the data from wide to long format

reshape long time internal external total age test, i(persnr) j(wave)

keep if total<.

*center gender

mcenter sex

*center age

mcenter age

*divide age by 10

replace C_age=C_age/10

*generate quadratic age

gen C_age2=C_age*C_age

*generate cubic age

gen C_age3=C_age*C_age*C_age

*center test

mcenter test

*center pastevent

mcenter pastevent

*mcenter life satisfaction

mcenter ls5

*standardize perceived control

zscore internal

zscore external

zscore total

*dichotomize age

gen C_agedi=0 if C_age<0 & C_age<.

replace C_agedi=1 if C_age>=0 & C_age<.

save control04, replace

//////////////////////////////////////////////////////////////

*selection and post-event difference effects

use control04, clear

*generate a variable "select"

*coded with 0 for assessments of perceived control in individuals who did not experience the respective loss

*coded with 1 for assessments of perceived control in individuals who experienced the respective loss -> before the respective loss

*coded with 2 for assessments of perceived control in individuals who experienced the respective loss -> in the month of and after the respective loss

gen select=0 if time==.

replace select=1 if time<0 & time<.

replace select=2 if time>=0 & time<.

*main effects

mixed z_internal C_sex C_age C_age2 C_age3 C_test C_pastevent ib0.select || hh: || persnr:,

mixed z_external C_sex C_age C_age2 C_age3 C_test C_pastevent ib0.select || hh: || persnr:,

mixed z_total C_sex C_age C_age2 C_age3 C_test C_pastevent ib0.select || hh: || persnr:,

*interactions with gender

mixed z_internal C_age C_age2 C_age3 C_test C_pastevent ib0.select##c.C_sex || hh: || persnr:,

mixed z_external C_age C_age2 C_age3 C_test C_pastevent ib0.select##c.C_sex || hh: || persnr:,

mixed z_total C_age C_age2 C_age3 C_test C_pastevent ib0.select##c.C_sex || hh: || persnr:,

*interactions with age

mixed z_internal C_sex C_age2 C_age3 C_test C_pastevent ib0.select##c.C_age || hh: || persnr:,

mixed z_external C_sex C_age2 C_age3 C_test C_pastevent ib0.select##c.C_age || hh: || persnr:,

mixed z_total C_sex C_age2 C_age3 C_test C_pastevent ib0.select##c.C_age || hh: || persnr:,

*death

*age*

sum age

sum age if C_agedi==0

sum age if C_agedi==1

*generate number of individuals

bysort persnr: gen byte person=(_n==1)

tab person if C_agedi==0

tab person if C_agedi==1

*interaction age * post-loss difference -> external control

mixed z_external C_sex C_age2 C_age3 C_test C_pastevent ib0.select##c.C_age || hh: || persnr:,

mixed z_external C_sex C_age2 C_age3 C_test C_pastevent ib0.select if C_agedi==0 || hh: || persnr:,

mixed z_external C_sex C_age2 C_age3 C_test C_pastevent ib0.select if C_agedi==1 || hh: || persnr:,

*interaction age * post-loss difference -> total control

mixed z_total C_sex C_age2 C_age3 C_test C_pastevent ib0.select##c.C_age || hh: || persnr:,

mixed z_total C_sex C_age2 C_age3 C_test C_pastevent ib0.select if C_agedi==0 || hh: || persnr:,

mixed z_total C_sex C_age2 C_age3 C_test C_pastevent ib0.select if C_agedi==1 || hh: || persnr:,

//////////////////////////////////////////////////////////////

*anticipation, socialization, short-term post-loss, and long-term post-loss effects

use control04, clear

keep if time<.

*generate a variable "longterm"

gen longterm=0 if time<=1

replace longterm=1 if time>1

*generate a variable "shortterm"

gen shortterm=0

replace shortterm=1 if time>0 & time<=1

*generate a variable "anticipation"

*coded with the time span between the assessment of perceived control and the respective loss for assessments of perceived control before the respective loss

*coded with 0 for assessments of perceived control in the month of and after the respective loss

gen ant=time

replace ant=0 if time>=0

*generate a variable "socialization"

*coded with the time span between the assessment of perceived control and the respective loss for assessments of perceived control after the respective loss

*coded with 0 for assessments of perceived control in the month of and before the respective loss

gen soc=time

replace soc=0 if time<=0

*generate interaction terms

*gender

gen sex_ant=C_sex*ant

gen sex_soc=C_sex*soc

gen sex_longterm=C_sex*longterm

gen sex_shortterm=C_sex*shortterm

*age

gen age_ant=C_age*ant

gen age_soc=C_age*soc

gen age_longterm=C_age*longterm

gen age_shortterm=C_age*shortterm

*life satisfaction

gen ls5_ant=C_ls5*ant

gen ls5_soc=C_ls5*soc

gen ls5_shortterm=C_ls5*shortterm

gen ls5_longterm=C_ls5*longterm

*main effects

mixed z_internal C_sex C_age C_age2 C_age3 C_test C_pastevent ant soc shortterm longterm || hh: || persnr:,

mixed z_external C_sex C_age C_age2 C_age3 C_test C_pastevent ant soc shortterm longterm || hh: || persnr:,

mixed z_total C_sex C_age C_age2 C_age3 C_test C_pastevent ant soc shortterm longterm || hh: || persnr:,

*main effects without quadratic and cubic age

mixed z_internal C_sex C_age C_test C_pastevent ant soc shortterm longterm || hh: || persnr:,

mixed z_external C_sex C_age C_test C_pastevent ant soc shortterm longterm || hh: || persnr:,

mixed z_total C_sex C_age C_test C_pastevent ant soc shortterm longterm || hh: || persnr:,

*interactions with gender

mixed z_internal C_sex C_age C_age2 C_age3 C_test C_pastevent ant soc shortterm longterm sex_ant || hh: || persnr:,

mixed z_internal C_sex C_age C_age2 C_age3 C_test C_pastevent ant soc shortterm longterm sex_soc || hh: || persnr:,

mixed z_internal C_sex C_age C_age2 C_age3 C_test C_pastevent ant soc shortterm longterm sex_shortterm || hh: || persnr:,

mixed z_internal C_sex C_age C_age2 C_age3 C_test C_pastevent ant soc shortterm longterm sex_longterm || hh: || persnr:,

mixed z_external C_sex C_age C_age2 C_age3 C_test C_pastevent ant soc shortterm longterm sex_ant || hh: || persnr:,

mixed z_external C_sex C_age C_age2 C_age3 C_test C_pastevent ant soc shortterm longterm sex_soc || hh: || persnr:,

mixed z_external C_sex C_age C_age2 C_age3 C_test C_pastevent ant soc shortterm longterm sex_shortterm || hh: || persnr:,

mixed z_external C_sex C_age C_age2 C_age3 C_test C_pastevent ant soc shortterm longterm sex_longterm || hh: || persnr:,

mixed z_total C_sex C_age C_age2 C_age3 C_test C_pastevent ant soc shortterm longterm sex_ant || hh: || persnr:,

mixed z_total C_sex C_age C_age2 C_age3 C_test C_pastevent ant soc shortterm longterm sex_soc || hh: || persnr:,

mixed z_total C_sex C_age C_age2 C_age3 C_test C_pastevent ant soc shortterm longterm sex_shortterm || hh: || persnr:,

mixed z_total C_sex C_age C_age2 C_age3 C_test C_pastevent ant soc shortterm longterm sex_longterm || hh: || persnr:,

*interactions with age

mixed z_internal C_sex C_age C_age2 C_age3 C_test C_pastevent ant soc shortterm longterm age_ant || hh: || persnr:,

mixed z_internal C_sex C_age C_age2 C_age3 C_test C_pastevent ant soc shortterm longterm age_soc || hh: || persnr:,

mixed z_internal C_sex C_age C_age2 C_age3 C_test C_pastevent ant soc shortterm longterm age_shortterm || hh: || persnr:,

mixed z_internal C_sex C_age C_age2 C_age3 C_test C_pastevent ant soc shortterm longterm age_longterm || hh: || persnr:,

mixed z_external C_sex C_age C_age2 C_age3 C_test C_pastevent ant soc shortterm longterm age_ant || hh: || persnr:,

mixed z_external C_sex C_age C_age2 C_age3 C_test C_pastevent ant soc shortterm longterm age_soc || hh: || persnr:,

mixed z_external C_sex C_age C_age2 C_age3 C_test C_pastevent ant soc shortterm longterm age_shortterm || hh: || persnr:,

mixed z_external C_sex C_age C_age2 C_age3 C_test C_pastevent ant soc shortterm longterm age_longterm || hh: || persnr:,

mixed z_total C_sex C_age C_age2 C_age3 C_test C_pastevent ant soc shortterm longterm age_ant || hh: || persnr:,

mixed z_total C_sex C_age C_age2 C_age3 C_test C_pastevent ant soc shortterm longterm age_soc || hh: || persnr:,

mixed z_total C_sex C_age C_age2 C_age3 C_test C_pastevent ant soc shortterm longterm age_shortterm || hh: || persnr:,

mixed z_total C_sex C_age C_age2 C_age3 C_test C_pastevent ant soc shortterm longterm age_longterm || hh: || persnr:,

*interactions with life satisfaction

mixed z_internal C_sex C_age C_age2 C_age3 C_test C_pastevent ant soc shortterm longterm C_ls5 ls5_ant || hh: || persnr:,

mixed z_internal C_sex C_age C_age2 C_age3 C_test C_pastevent ant soc shortterm longterm C_ls5 ls5_soc || hh: || persnr:,

mixed z_internal C_sex C_age C_age2 C_age3 C_test C_pastevent ant soc shortterm longterm C_ls5 ls5_shortterm || hh: || persnr:,

mixed z_internal C_sex C_age C_age2 C_age3 C_test C_pastevent ant soc shortterm longterm C_ls5 ls5_longterm || hh: || persnr:,

mixed z_external C_sex C_age C_age2 C_age3 C_test C_pastevent ant soc shortterm longterm C_ls5 ls5_ant || hh: || persnr:,

mixed z_external C_sex C_age C_age2 C_age3 C_test C_pastevent ant soc shortterm longterm C_ls5 ls5_soc || hh: || persnr:,

mixed z_external C_sex C_age C_age2 C_age3 C_test C_pastevent ant soc shortterm longterm C_ls5 ls5_shortterm || hh: || persnr:,

mixed z_external C_sex C_age C_age2 C_age3 C_test C_pastevent ant soc shortterm longterm C_ls5 ls5_longterm || hh: || persnr:,

mixed z_total C_sex C_age C_age2 C_age3 C_test C_pastevent ant soc shortterm longterm C_ls5 ls5_ant || hh: || persnr:,

mixed z_total C_sex C_age C_age2 C_age3 C_test C_pastevent ant soc shortterm longterm C_ls5 ls5_soc || hh: || persnr:,

mixed z_total C_sex C_age C_age2 C_age3 C_test C_pastevent ant soc shortterm longterm C_ls5 ls5_shortterm || hh: || persnr:,

mixed z_total C_sex C_age C_age2 C_age3 C_test C_pastevent ant soc shortterm longterm C_ls5 ls5_longterm || hh: || persnr:,

*main models including random effects

mixed z_internal C_sex C_age C_age2 C_age3 C_test C_pastevent ant soc shortterm longterm || hh: || persnr: ant soc shortterm longterm

mixed z_external C_sex C_age C_age2 C_age3 C_test C_pastevent ant soc shortterm longterm || hh: || persnr: ant soc shortterm longterm

mixed z_total C_sex C_age C_age2 C_age3 C_test C_pastevent ant soc shortterm longterm || hh: || persnr: ant soc shortterm longterm

*separation

*gender

*interaction gender * short-term post-loss -> external control

mixed z_external C_sex C_age C_age2 C_age3 C_test C_pastevent ant soc shortterm longterm sex_shortterm || hh: || persnr:,

mixed z_external C_age C_age2 C_age3 C_test C_pastevent ant soc shortterm longterm if sex==0 || hh: || persnr:,

mixed z_external C_age C_age2 C_age3 C_test C_pastevent ant soc shortterm longterm if sex==1 || hh: || persnr:,

*age

mcenter C_age

gen help=0 if C_C_age<0 & C_C_age<.

replace help=1 if C_C_age>=0 & C_C_age<.

sum age

sum age if help==0

sum age if help==1

*generate number of individuals

bysort persnr: gen byte person=(_n==1)

tab person if help==0

tab person if help==1

*interaction age * short-term post-loss -> internal control

mixed z_internal C_sex C_age C_age2 C_age3 C_test C_pastevent ant soc shortterm longterm age_shortterm || hh: || persnr:,

mixed z_internal C_sex C_age2 C_age3 C_test C_pastevent ant soc shortterm longterm if help==0 || hh: || persnr:,

mixed z_internal C_sex C_age2 C_age3 C_test C_pastevent ant soc shortterm longterm if help==1 || hh: || persnr:,

*death

*age

mcenter C_age

gen help=0 if C_C_age<0 & C_C_age<.

replace help=1 if C_C_age>=0 & C_C_age<.

sum age

sum age if help==0

sum age if help==1

*generate number of individuals

bysort persnr: gen byte person=(_n==1)

tab person if help==0

tab person if help==1

*interaction age * long-term post-loss -> external control

mixed z_external C_sex C_age C_age2 C_age3 C_test C_pastevent ant soc shortterm longterm age_longterm || hh: || persnr:,

mixed z_external C_sex C_age2 C_age3 C_test C_pastevent ant soc shortterm longterm if help==0 || hh: || persnr:,

mixed z_external C_sex C_age2 C_age3 C_test C_pastevent ant soc shortterm longterm if help==1 || hh: || persnr:,

*interaction age * long-term post-loss -> total control

mixed z_total C_sex C_age C_age2 C_age3 C_test C_pastevent ant soc shortterm longterm age_longterm || hh: || persnr:,

mixed z_total C_sex C_age2 C_age3 C_test C_pastevent ant soc shortterm longterm if help==0 || hh: || persnr:,

mixed z_total C_sex C_age2 C_age3 C_test C_pastevent ant soc shortterm longterm if help==1 || hh: || persnr:,

//////////////////////////////////////////////////////////////

*sample characteristics

*internal consistencies per year

use control02, clear

*total

alpha kp7101 kp7103 kp7106 kp7102r kp7104r kp7105r kp7107r kp7108r

alpha lp0801 lp0803 lp0806 lp0802r lp0804r lp0805r lp0807r lp0808r

alpha mp1001 mp1003 mp1006 mp1002r mp1004r mp1005r mp1007r mp1008r

*int

alpha kp7101 kp7103 kp7106

alpha lp0801 lp0803 lp0806

alpha mp1001 mp1003 mp1006

*ext

alpha kp7102 kp7104 kp7105 kp7107 kp7108

alpha lp0802 lp0804 lp0805 lp0807 lp0808

alpha mp1002 mp1004 mp1005 mp1007 mp1008

*averages internal consistencies across all three waves

gen cronbach_total=(0.7092+0.7251+0.7261)/3

tab cronbach_total

gen cronbach_internal=(0.5629+0.5580+0.5602)/3

tab cronbach_internal

gen cronbach_external=(0.7682+0.7687+0.7763)/3

tab cronbach_external

*sample characteristics

use control03, clear

for any 94 95 96: tab sex if totalX<.

for any 94 95 96: gen assessX=0

for any 94 95 96: replace assessX=1 if totalX<.

gen number=assess94+assess95+assess96

tab event_y

tab event_y assess94, row

tab event_y assess95, row

tab event_y assess96, row

tabstat number, by(event_y) stat(mean sd)

tab eventdi assess94, row

tab eventdi assess95, row

tab eventdi assess96, row

tabstat number, by(eventdi) stat(mean sd)

*sample characteristics

use control03, clear

tab sex eventdi, col

tab pastevent eventdi, col

gen numbereventdi=0

replace numbereventdi=1 if numberevent>1 & numberevent<.

tab numbereventdi eventdi, col

use control04, clear

sum age if eventdi==0

sum age if eventdi==1

sum internal if eventdi==0

sum internal if eventdi==1

sum external if eventdi==0

sum external if eventdi==1

sum total if eventdi==0

sum total if eventdi==1

//////////////////////////////////////////////////////////////

*VIF

use control04, clear

gen select=0 if time==.

replace select=1 if time<0 & time<.

replace select=2 if time>=0 & time<.

*vif

regress z_internal C_sex C_age C_age2 C_age3 C_test C_pastevent ib0.select

vif

regress z_external C_sex C_age C_age2 C_age3 C_test C_pastevent ib0.select

vif

regress z_total C_sex C_age C_age2 C_age3 C_test C_pastevent ib0.select

vif

keep if time<.

gen longterm=0 if time<=1

replace longterm=1 if time>1

gen shortterm=0

replace shortterm=1 if time>0 & time<=1

gen ant=time

replace ant=0 if time>=0

gen soc=time

replace soc=0 if time<=0

*vif

regress z_internal C_sex C_age C_age2 C_age3 C_test C_pastevent ant soc shortterm ib0.longterm

vif

regress z_external C_sex C_age C_age2 C_age3 C_test C_pastevent ant soc shortterm ib0.longterm

vif

regress z_total C_sex C_age C_age2 C_age3 C_test C_pastevent ant soc shortterm ib0.longterm

vif

//////////////////////////////////////////////////////////////

*LOESS curves

*separation

cd "XXXControl/separation/"

use control04, clear

keep if time>=-5 & time<=5

twoway lowess z_internal time, bwidth(0.8)

graph save "Graph" internal.gph, replace

twoway lowess z_external time, bwidth(0.8)

graph save "Graph" external.gph, replace

twoway lowess z_total time, bwidth(0.8)

graph save "Graph" total.gph, replace

*death of a partner

cd "XXXControl/death/"

use control04, clear

keep if time>=-5 & time<=5

twoway lowess z_internal time, bwidth(0.8)

graph save "Graph" internal.gph, replace

//////////////////////////////////////////////////////////////

*partners per household

cd "XXXSOEP_v35/"

use pgen, clear

mvdecode pgpartz, mv(-1 -2 -3 -4 -5 -6 -7 -8 -9)

gen wave=.

for any 94 95 96: replace wave=X if syear==19X

keep pgpartz pid wave

keep if wave<.

rename pid persnr

reshape wide pgpartz, i(persnr) j(wave)

cd "XXXControl/death/"

save part, replace

use control03, clear

merge persnr using part, sort

keep if _merge==3

gen part=pgpartz94

replace part=pgpartz95 if total94==.

replace part=pgpartz96 if total94==. & total95==.

*generate a variable "partner"

*coded with 0 for individuals who WERE NOT living with a partner in the same household

*coded with 1 for individuals who WERE living with a partner in the same household

gen partner=.

replace partner=0 if part==0

replace partner=1 if part==1 | part==2 | part==3 | part==4 | part==5

tab part

tab partner

*generate number of individuals

bysort hh persnr: gen byte person=(_n==1)

*calculate number of persons per household

egen totpeople=sum(person), by(hh)

tab totpeople

tab partner if totpeople>1

keep if eventdi==1

capture drop person

capture drop totpeople

*generate number of individuals

bysort hh persnr: gen byte person=(_n==1)

*generate number of individuals per household

egen totpeople=sum(person), by(hh)

tab totpeople

tab partner if totpeople>1

use control04, clear

mixed z_total C_sex || hh: || persnr:,

keep if time<.

mixed z_total C_sex || hh: || persnr:,

//////////////////////////////////////////////////////////////

*model fit

cd "XXXControl/death/"

use "XXXControl/death/control04.dta", clear

keep if time<.

*generate a variable "longterm"

gen longterm=0 if time<=1

replace longterm=1 if time>1

*generate a variable "shortterm"

gen shortterm=0

replace shortterm=1 if time>0 & time<=1

*generate a variable "anticipation"

*coded with the time span between the assessment of perceived control and the respective loss for assessments of perceived control before the respective loss

*coded with 0 for assessments of perceived control in the month of and after the respective loss

gen ant=time

replace ant=0 if time>=0

*generate a variable "socialization"

*coded with the time span between the assessment of perceived control and the respective loss for assessments of perceived control after the respective loss

*coded with 0 for assessments of perceived control in the month of and before the respective loss

gen soc=time

replace soc=0 if time<=0

*internal

*1

mixed z_internal || hh: || persnr:,

estimates store one

mixed z_internal C_sex || hh: || persnr:,

estimates store two

lrtest two one

*2

mixed z_internal C_sex || hh: || persnr:,

estimates store one

mixed z_internal C_sex C_age || hh: || persnr:,

estimates store two

lrtest two one

*3

mixed z_internal C_sex C_age || hh: || persnr:,

estimates store one

mixed z_internal C_sex C_age C_age2 C_age3 || hh: || persnr:,

estimates store two

lrtest two one

*4

mixed z_internal C_sex C_age C_age2 C_age3 || hh: || persnr:,

estimates store one

mixed z_internal C_sex C_age C_age2 C_age3 C_test || hh: || persnr:,

estimates store two

lrtest two one

*5

mixed z_internal C_sex C_age C_age2 C_age3 C_test || hh: || persnr:,

estimates store one

mixed z_internal C_sex C_age C_age2 C_age3 C_test C_pastevent ant soc shortterm longterm || hh: || persnr:,

estimates store two

lrtest two one

*external

*1

mixed z_external || hh: || persnr:,

estimates store one

mixed z_external C_sex || hh: || persnr:,

estimates store two

lrtest two one

*2

mixed z_external C_sex || hh: || persnr:,

estimates store one

mixed z_external C_sex C_age || hh: || persnr:,

estimates store two

lrtest two one

*3

mixed z_external C_sex C_age || hh: || persnr:,

estimates store one

mixed z_external C_sex C_age C_age2 C_age3 || hh: || persnr:,

estimates store two

lrtest two one

*4

mixed z_external C_sex C_age C_age2 C_age3 || hh: || persnr:,

estimates store one

mixed z_external C_sex C_age C_age2 C_age3 C_test || hh: || persnr:,

estimates store two

lrtest two one

*5

mixed z_external C_sex C_age C_age2 C_age3 C_test || hh: || persnr:,

estimates store one

mixed z_external C_sex C_age C_age2 C_age3 C_test C_pastevent ant soc shortterm longterm || hh: || persnr:,

estimates store two

lrtest two one

*total

*1

mixed z_total || hh: || persnr:,

estimates store one

mixed z_total C_sex || hh: || persnr:,

estimates store two

lrtest two one

*2

mixed z_total C_sex || hh: || persnr:,

estimates store one

mixed z_total C_sex C_age || hh: || persnr:,

estimates store two

lrtest two one

*3

mixed z_total C_sex C_age || hh: || persnr:,

estimates store one

mixed z_total C_sex C_age C_age2 C_age3 || hh: || persnr:,

estimates store two

lrtest two one

*4

mixed z_total C_sex C_age C_age2 C_age3 || hh: || persnr:,

estimates store one

mixed z_total C_sex C_age C_age2 C_age3 C_test || hh: || persnr:,

estimates store two

lrtest two one

*5

mixed z_total C_sex C_age C_age2 C_age3 C_test || hh: || persnr:,

estimates store one

mixed z_total C_sex C_age C_age2 C_age3 C_test C_pastevent ant soc shortterm longterm || hh: || persnr:,

estimates store two

lrtest two one
